# Nitrogen-dependent regulation of nitrate reductase in coral symbioses

**DOI:** 10.1093/ismejo/wrag260

**Published:** 2026-10-08

**Authors:** Chloé Stévenne, Christine Ferrier-Pagès, Renaud Grover, Jean-Christophe Plumier, Stéphane Roberty

**Affiliations:** Animal Physiology, InBioS, University of Liège, 4 Chemin de la Vallée, 4000 Liège, Belgium; Coral Ecophysiology Team, Centre Scientifique de Monaco, 8 Quai Antoine 1er, MC 98000, Monaco; Coral Ecophysiology Team, Centre Scientifique de Monaco, 8 Quai Antoine 1er, MC 98000, Monaco; Animal Physiology, InBioS, University of Liège, 4 Chemin de la Vallée, 4000 Liège, Belgium; Animal Physiology, InBioS, University of Liège, 4 Chemin de la Vallée, 4000 Liège, Belgium

**Keywords:** coral symbiosis, Symbiodiniaceae, nitrate reductase, nitrogen assimilation, nitrogen metabolism, enzyme regulation, post-transcriptional regulation, light-dependent regulation

## Abstract

The nutritional symbiosis between corals and their photosynthetic dinoflagellate partners underpins the ecological success of reef-building corals in nutrient-poor environments. Although coral holobionts can assimilate the abundant yet highly variable environmental nitrate, direct insight into how nitrate reductase is regulated in these symbiotic algae has been lacking. Here, nitrate reductase protein abundance and gene expression were characterized in cultured Symbiodiniaceae exposed to different nitrogen regimes and light conditions, revealing the multifactorial nature of its regulation. The results indicate that nitrate reductase behaves as a substrate-induced enzyme: nitrate stimulates protein synthesis in nitrogen-starved cultures, whereas ammonium actively suppresses its expression in a concentration-dependent manner. Light availability and photosynthetic electron transport further modulate protein abundance, suggesting that although nitrate reductase synthesis depends on nitrate availability, its stability may rely on photosynthesis. *In hospite*, nitrate reductase is synthesized within hours of nitrate exposure by symbionts from nitrogen-starved corals, indicating that nitrate reduction can occur within the host environment. However, this response is transient and reduced relative to free-living cells, suggesting that nitrate reduction may function as a facultative pathway activated when preferred nitrogen sources become limited. Finally, gene expression measurements and pharmacological inhibition experiments are consistent with predominant post-transcriptional regulation of nitrate reductase protein expression, enabling rapid and reversible control of nitrate assimilation. Together, these findings describe a tightly regulated and responsive nitrate reduction system in coral symbionts that provides a flexible mechanism contributing to nitrogen homeostasis under fluctuating nutrient regimes.

## Introduction

Coral reefs are among the most biologically diverse and productive ecosystems on Earth, yet they thrive in some of the ocean’s most oligotrophic waters. This paradox is largely resolved by the intimate symbiosis between scleractinian corals and their photosynthetic dinoflagellates of the family Symbiodiniaceae, which, together with other associated microorganisms, constitute a holobiont [1]. Symbiodiniaceae provide their coral hosts with photosynthetically derived carbohydrates, amino acids, and lipids, thereby supporting the host’s metabolism and growth in nutrient-poor environments. *In hospite*, the algae receive inorganic nutrients and metabolic waste products from the host and benefit from the protective environment of the symbiosome [2, 3]. Although corals are also capable of heterotrophic feeding [4], their survival and ecological success are primarily sustained by their symbionts’ autotrophic productivity [5, 6].

Among the key nutrients required for this partnership, nitrogen is particularly critical due to its role in the synthesis of proteins and other essential biomolecules. In marine environments, nitrogen is present in several forms, with nitrate (NO_3_^−^) being the most abundant source of bioavailable inorganic nitrogen [7, 8]. Coral holobionts preferentially assimilate ammonium (NH_4_^+^) because of its reduced state and the lower energetic cost of assimilation [9, 10]. Both the host and symbionts assimilate NH_4_^+^ via their glutamine synthetase/glutamate dehydrogenase (GS/GDH) and glutamine synthetase-glutamate synthase (GS-GOGAT) pathways. In contrast, coral hosts lack nitrate and nitrite reductase (NR and NiR), preventing them from utilizing NO_3_^−^ directly [11–13].

Due to its environmental abundance, NO_3_^−^ remains a potentially important nitrogen source through assimilation by Symbiodiniaceae [10, 14]. Experimental studies have shown that coral holobionts can remove NO_3_^−^ from the surrounding water [10, 15, 16] and incorporate isotopically labelled nitrate into both algal and host tissues [14, 17, 18]. However, the conditions under which nitrate assimilation is induced or repressed, as well as the molecular and enzymatic mechanisms that underpin this process in Symbiodiniaceae, remain largely uncharacterized. Early enzymatic assays indicated that NR activity is induced by nitrate and light but is suppressed by ammonium [15, 19]. Although NR activity was initially measured in freshly isolated symbionts (FIS) [15], it could not be reproduced in subsequent experiments on FIS or *in hospite* symbionts [20], leading to the hypothesis that host-derived NH_4_^+^ represses NR expression [19, 21]. Since then, direct assessments of NR activity or regulation in Symbiodiniaceae have been lacking, and the inhibitory role of NH_4_^+^ remains unresolved. Similarly, evidence for a role of light remains equivocal, as subsequent studies examined only NO_3_^−^ uptake, rather than NR expression or activity, in cultured Symbiodiniaceae [9], FIS [22], or corals [10, 16] with inconsistent outcomes. Consequently, the roles of light and photosynthesis in regulating NR in Symbiodiniaceae remain unconfirmed.

Investigations have primarily relied on transcriptomics and gene predictions to examine the expression of nitrate assimilation-related proteins such as nitrate transporters (NRTs), nitrate and nitrite reductase or GS-GOGAT proteins [23–26]. Although these studies confirm the genetic potential for nitrate assimilation, they provide limited insight into NR protein abundance, enzyme activity, or post-transcriptional regulation.

The intracellular location of symbionts poses an additional layer of complexity for nitrate acquisition. At physiological pH, NO₃^−^ is charged and thus requires active transport across lipid membranes [21]. Although NRTs have been identified in Symbiodiniaceae [24, 27–29], the mechanism by which nitrate is transported across the host cell membrane into the symbiosome remains unclear. The only indication of a potential transport pathway is the identification of SIALIN-like sequences in the genomes of several coral species, possibly homologous to the H^+^:2NO_3_^−^ cotransporter SIALIN [30].

Understanding nitrate assimilation in coral symbioses requires direct characterization of nitrate reduction regulation at both the molecular and cellular levels. Such insight is essential for determining how corals manage nitrogen resources, particularly under changing environmental conditions that may alter nutrient availability. In this study, we investigated the expression patterns of nitrate reductase in Symbiodiniaceae, both in culture and *in hospite*. Using western blotting (WB) and quantitative reverse transcription polymerase chain reaction (RT-qPCR), we characterized NR protein and gene expression in response to different nitrogen sources (NO_3_^−^ and NH_4_^+^) and concentrations and examined the influence of light and photosynthetic electron flow. In addition, we tested the effects of various inhibitors to shed light on potential regulatory mechanisms at the transcriptional and post-transcriptional levels. Finally, we examined NR protein expression in symbionts from three coral species following NO_3_^−^ enrichment, providing insight into nitrate assimilation within the coral-algal symbiosis.

## Materials and methods

### Symbiodiniaceae strains and culture conditions

*Breviolum minutum* (formerly *Symbiodinium minutum*) strain SSB01, a member of Symbiodiniaceae, was used for all culture-based experiments. In addition, the first culture-based experiment was repeated on a *Symbiodinium microadriaticum* strain RSStyP-A for comparative WB analysis. SSB01 was originally isolated from the sea anemone *Exaiptasia diaphana* line H2 in the Pringle laboratory (Stanford University, USA) [31], whereas RSStyP-A was isolated from a colony of the Red Sea coral *Stylophora pistillata* in the Levy laboratory (Bar Ilan University, Israel). Both strains were kindly provided by Guse lab (LMU Munich, Germany) and Levy lab, respectively. Strain identities were confirmed by ITS2 rRNA gene sequencing as described in Supporting Information S1.

Symbiodiniaceae strains were grown and maintained in a Percival I-36LLX incubator (Percival Scientific Inc, USA) at 26°C and under 150 μmol photon m^−2^ s^−1^ on a 12 h:12 h light:dark cycle provided by cool white 10 000 K (Sylvania Aquastar F18W/174-T8) and blue 20 000°k lights (Aquavie Lumivie SM T8). Symbiodiniaceae cells were maintained in a modified f/2 medium (Supporting Information S2-S4) [32, 33] made in artificial seawater (ASW) at a salinity of 34 prepared with Coral Pro Salt (Red Sea Aquatics, UK). Each strain was grown with 500 μM of nitrogen either in the form of NO_3_^−^ or NH_4_^+^ and were kept in triplicate culture flasks for each condition in volumes ranging from 150 ml to 300 ml. Exponential growth was maintained by subculturing into fresh medium once a week. All experiments were conducted during the exponential growth phase of the cultures.

### Experimental treatments applied to Symbiodiniaceae in cultures

Five experimental treatments were conducted to elucidate the factors influencing NR expression in Symbiodiniaceae strains (Fig. 1). Prior to treatments, cultures grown under long-term (LT) conditions in 500 μM NO_3_^−^ f/2 medium were transitioned to a nitrogen-free (NF) f/2 medium for 4 days to deplete cellular nitrogen levels. This approach was adopted following observations that NR protein was either undetectable or present at very low and inconsistent levels in nitrogen-replete algae (Supporting Information S5). Nitrogen depletion ensured a consistently low NR baseline and standardized initial conditions prior to treatment incubations.

**Figure 1:**
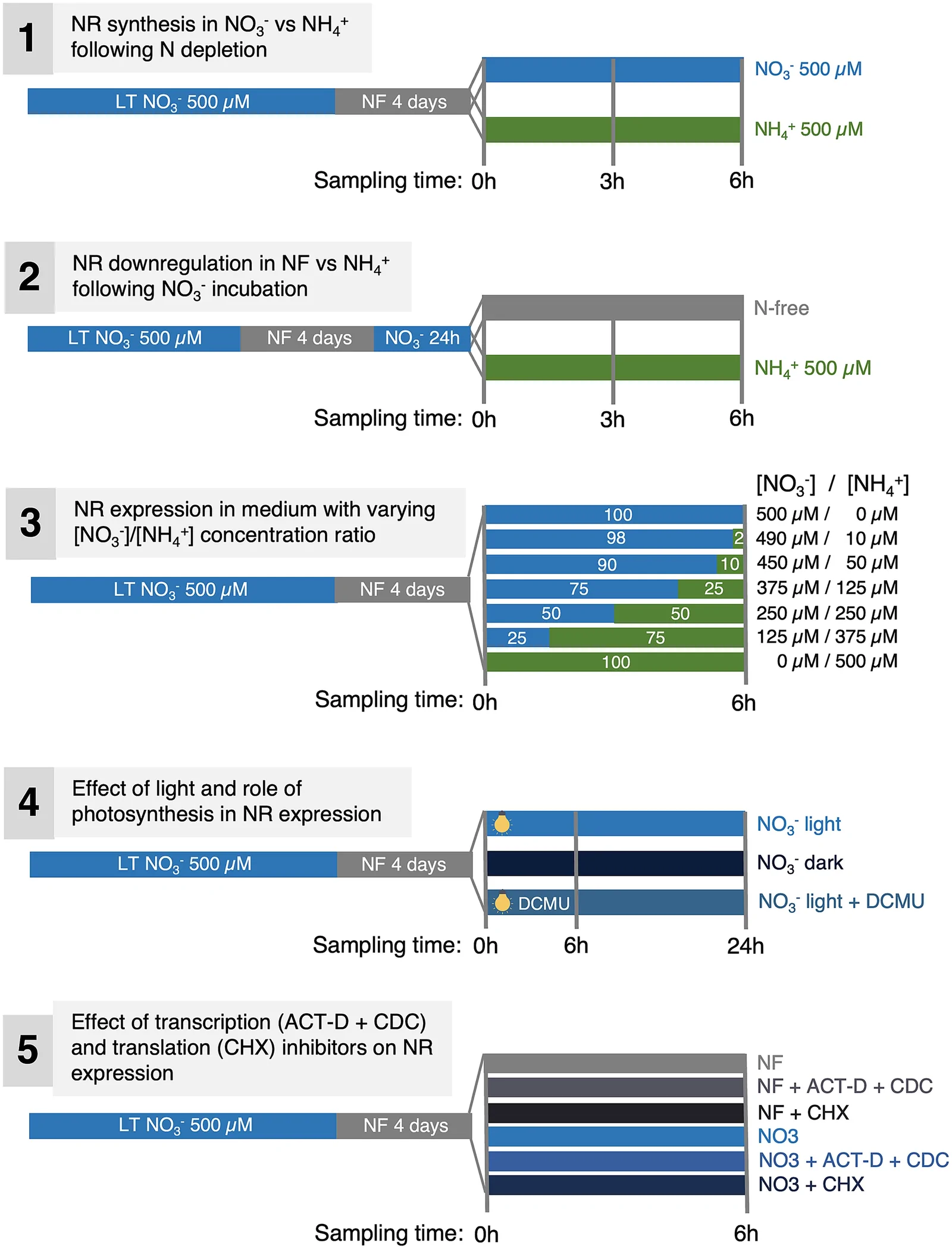
Summary of experimental conditions of the five experiments investigating nitrate reductase expression and regulation in Symbiodiniaceae strains in cultures. Treatment conditions are schematically represented by colour bands corresponding to the incubation interval and the nitrogen treatment. The colours are associated with the nitrogen treatment with grey for NF, blue for nitrate, and green for ammonium. Special treatments such as the addition of inhibitors and light treatments are represented by darker shades of grey or blue. The total nitrogen concentration in treatment media was always set to 500 μM. Experiments 1,2, and 3: Symbiodiniaceae cells were transitioned between the different nitrogen treatments (NF, NO_3_^−^, NH_4_^+^, and mixtures of NO_3_^−^ and NH_4_^+^) and incubated for 3 to 6 h. Experiment 4: 12 h dark-incubated (night cycle) Symbiodiniaceae were transitioned under one of three conditions and incubated for 6 to 24 h: control light (150 μmol photons m^−2^ s^−1^), complete darkness (0 μmol photons m^−2^ s^−1^), or standard light with 40 μM of DCMU, a PSII inhibitor. Experiment 5: Symbiodiniaceae cells were placed under six experimental conditions and incubated for 6 h. These conditions combined two nitrogen treatments—NF or NO_3_^−^ 500 μM—with three inhibitor treatments: no inhibitors, transcription inhibitors, or translation inhibitors. Transcription inhibitors ACT-D and CDC were added together at final concentrations of 0.5 μM and 20 μg/ml, respectively [34], whereas translation inhibitor CHX was added at a final concentration of 10 μg/ml [35, 36].

Experiments 1 and 2 aimed to identify the individual effects of the nitrogen source on NR expression, whereas Experiment 3 examined NR expression in response to different concentration ratios of NO_3_^−^ and NH_4_^+^. Experiment 4 investigated the influence of light and photosynthesis on NR protein and gene expression. Experiment 5 explored the roles of transcriptional and post-transcriptional regulation in NR expression using the transcription inhibitors actinomycin-D (ACT-D) and cordycepin (CDC), as well as the translation inhibitor cycloheximide (CHX) [34–36].

### NR protein expression in cultured Symbiodiniaceae

At each sampling time (Fig. 1), 15 ml of Symbiodiniaceae culture (15–30 mg fresh weight) were collected per replicate. Protein extracts were prepared using a Tris-base extraction buffer with a protease inhibitor cocktail and cell disruption using 250 μl of glass beads (710–1180 nm, acid-washed, Sigma-Aldrich) in a TissueLyser II (QIAGEN) for 10 min at 30 Hz. Protein concentrations were determined by spectrophotometry using the colorimetric RC DC Protein Assay (BioRad, USA).

The protein extracts were then prepared for WB together with a sample of purified nitrate reductase isolated from *Arabidopsis thaliana* (N0163-1VL, Sigma-Aldrich, Merck, Germany), which was used as an immunoreactive positive control. Denatured protein extracts in Laemmli buffer [37] were separated by electrophoresis on an SDS-PAGE gel (80 V for 30 min followed by 140 V for 60 min) and transferred to a polyvinylidene difluoride membrane (Immun-Blot PVDF, Bio-Rad, USA). Primary antibodies for nitrate reductase detection (dilution 1/1000, Assimilatory NR, Agrisera AS08 310) were incubated first and primary antibodies for Cyt f detection (dilution 1/5000, algal cytochrome f PetA of the thylakoid Cyt b6/f-complex, Agrisera AS06 119), used as a protein loading control, were incubated the next day after a first visualization of NR proteins.

Immunoreactive bands were detected with horseradish peroxidase (HRP) conjugated to anti-rabbit secondary antibodies (dilution 1/25 000, goat anti-rabbit IgG HRP conjugated, Agrisera AS09 602) and using the Amersham ECL Prime detection system (Cytiva, USA) on an iBright FL1000 imaging system (Thermo Scientific, USA). Analysis and quantification of immunoblot bands were performed using the iBright analysis software (Thermo Scientific, USA). Nitrate reductase protein expression was calculated as the ratio between the consistently detected ~110 kDa NR-immunoreactive band and Cyt f band signal expressed as local background corrected volume (Supporting Information S6). To validate the identity of the nitrate reductase band detected by WB, protein extracts from four independent *B. minutum* cultures were separated by SDS-PAGE. For each culture, the gel region corresponding to the NR-immunoreactive band was excised and analysed by liquid chromatography tandem mass spectrometry (LC–MS/MS; Vanquish Neo UHPLC system coupled to an Orbitrap Fusion Lumos Tribrid mass spectrometer, Thermo Fisher Scientific, USA). Protein identification was performed using two independent *B. minutum*-related protein sequence databases constructed from genome [38] and transcriptome [39–41] gene models (Supporting Information S7). Antibody recognition was additionally evaluated by peptide competition using the authentic immunizing peptide supplied by the antibody manufacturer (Agrisera) and recombinant *A. thaliana* nitrate reductase as a positive control (Supporting Information S8).

### *NR* gene expression in cultured Symbiodiniaceae

*Nitrate reductase* gene expression was investigated in SSB01 samples from Experiments 1, 2, 4, and 5 (Fig. 1) using quantitative RT-qPCR. At each sampling time, 15 ml of SSB01 culture (15–30 mg fresh weight) was sampled from each replicate in each experimental condition. RNA was extracted from the algae using the Purelink RNA Mini Kit (Thermo Scientific, USA) according to the manufacturer’s instructions. Cell disruption was achieved in 0.9 ml of lysis buffer with acid-washed glass beads (710–1180 μm, Sigma-Aldrich) using bead-beating on the TissueLyser II (QIAGEN) for 7 min at 30 Hz. RNA was precipitated with absolute molecular grade ethanol, purified on spin columns including DNase treatment, and eluted in 25 μl RNase-free water. Total RNA concentrations were quantified, and RNA quality was assessed by spectrophotometry on a Take3 microspot plate using a Synergy Mx BioTek plate reader. cDNA was synthetized from 0.25 to 1 μg RNA (varying by experiment but consistent within each) by reverse transcription using the RevertAid H Minus First Strand Kit (Thermo Scientific, USA), with a 60 min incubation at 42°C followed by 5 min at 70°C.

The primer pairs Cyc_Maru [42] and Act1_May [43] for genes *cyclophilin* and *actin1*, respectively, were selected as housekeeping genes (HKG) (Supporting Information S9). The nitrate reductase primer pair NR_s6_34 was selected on the putative *NR* transcript s6_34 as previously described [24]. This primer pair has previously been used to examine *NR* gene expression levels in SSB01 cultures and in symbionts isolated from the sea anemone *E. diaphana* [42]. Primer sequences and specifications are summarized in Table 1.

**Table 1:** Primer pair names, sequences, and amplicon sizes used for RT-qPCR of the gene of interest (NR) and housekeeping genes (Cyc and Act1) in *B. minutum* SSB01.

Gene	Primer pair name	Forward primer (5′-3′)	Reverse primer (5′-3′)	Amplicon size	Ref.
*Nitrate reductase*	NR_s6_34	TGATGATGTATTCGGGTTTG	TCAGACAAGCCTTCGGAAAATC	118 bp	[24]
*Cyclophilin*	Cyc_Maru	TGCTTGAGGGTAAAGTTCTC	CAACCCCTTCATTTCAAAGG	Undetermined (~100 bp)	[42]
*Actin 1*	Act1_May	CACCACCATGTTCACAGGAA	AGCCACCACCTTGATCTTCA	87 bp	[43]

Primer sequences and amplicon sizes were obtained from the literature.

Quantitative reverse transcription polymerase chain reactions (RT-qPCR) were performed in technical triplicate for each sample using 4 μl of 1:50 diluted templates in 10 μl reaction volumes with Takyon Low ROX SYBR 2× MasterMix blue dTTP (Eurogentec, Belgium). RT-qPCRs were performed on a QuantStudio 5 system (Thermo Scientific, USA) according to a PCR profile consisting of an initial incubation of 2 min at 50°C and 2 min at 95°C, followed by 40 cycles of 15 s at 95°C, and 1 min at 60°C. Runs were terminated with melt curves from 60°C to 95°C, and RNase-free water controls were included as negative controls. Relative expression of genes was expressed as ∆∆Ct and fold gene expression as 2^-∆∆Ct^ (Supporting Information S10). Statistical analyses were performed on ∆∆Ct values using ANOVA and *post hoc* Tukey’s honestly significant difference (HSD) tests.

### NR expression in *in hospite* Symbiodiniaceae

Nitrate reductase expression by Symbiodiniaceae was investigated *in hospite* in *S. pistillata* (Esper 1792), *Turbinaria reniformis* (Bernard 1896), and *Echinopora lamellosa* (Esper 1791), and their FIS. These three coral species from different families were selected for their association with diverse symbiont assemblages [44–49], and their different trophic and nutritional capabilities [49–52]. Coral colonies were maintained in flow-through seawater aquaria at the Centre Scientifique de Monaco (Monaco). Nubbins were produced from mother colonies, evenly distributed between two independent tanks, and allowed to recover under controlled environmental conditions. During the 4-week recovery, they were fed twice weekly with *Artemia* nauplii, and feeding was stopped 2 weeks before experiments to prevent interactions with nitrogen enrichments (Supporting Information S11).

Two experiments were performed to investigate NR protein expression in Symbiodiniaceae *in hospite* (Experiment A performed on all three coral species) and in Symbiodiniaceae freshly isolated (FIS) from their coral host (Experiment B performed on FIS from *S. pistillata* and *T. reniformis*) (Fig. 2). For both experiments, coral nubbins were first incubated in nitrogen-depleted water for 24 h (depletion of dissolved inorganic nitrogen, DIN, to ≤0.08 μM based on previously validated methodology [53]; Supporting Information S11).

**Figure 2:**
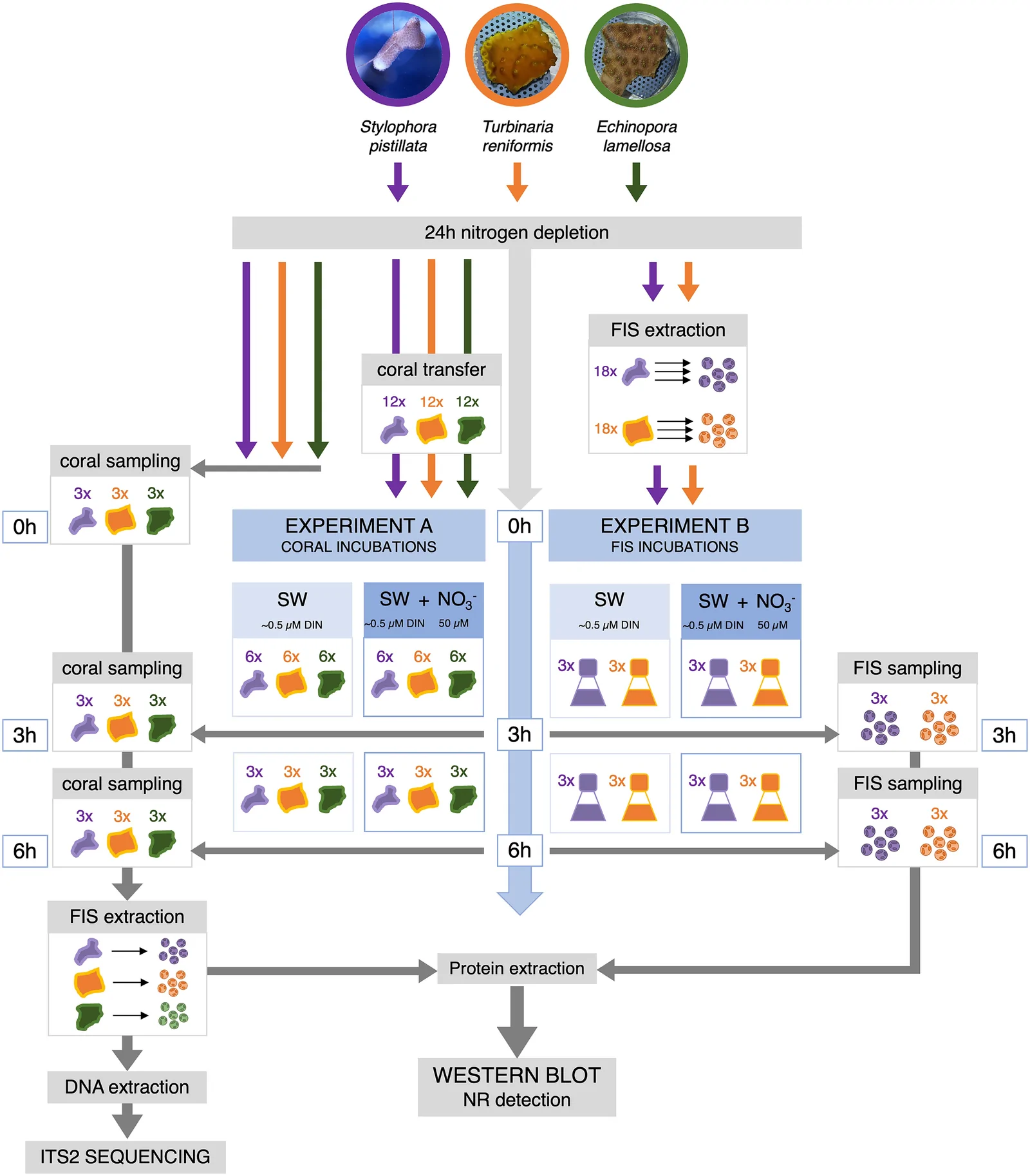
Schematic overview of the experimental design used to investigate nitrate reductase protein expression in symbionts *in hospite* (Experiment A) and in FIS (Experiment B) of the corals *S. pistillata*, *T. reniformis*, and *E. lamellosa* (only Experiment A). Corals were first subjected to 24 h nitrogen depletion and sampled at 0 h prior to transfer into experimental treatments. Control conditions consisted of FSW naturally containing ~0.5–0.7 μM DIN and 0.2 μM DIP. Nitrate-enriched conditions consisted of the same FSW supplemented with 50 μM NO_3_^−^. (A) Coral nubbins were incubated in duplicate 28 L tanks maintained at 25°C ± 0.5°C, under a 12 h:12 h light:dark cycle at 150 μmol photons m^−2^ s^−1^, with continuous water circulation. Tanks were operated as closed systems, and nitrate enrichment was achieved by adding a NaNO_3_ stock solution immediately prior to incubation. (B) FIS were extracted from N-depleted corals and incubated in 200 ml ventilated culture flasks at 25°C ± 0.5°C under 150 μmol photons m^−2^ s^−1^ constant light with gentle agitation using a magnetic stirrer. Samples were collected at 0, 3, and 6 h of incubation time for downstream NR protein analysis (WB) and ITS2 sequencing analysis.

For Experiment A, six nubbins per coral species were transferred from N-depleted seawater into each treatment. Control nubbins were incubated for 6 h in natural oligotrophic filtered seawater (FSW, ~0.5–0.7 μM DIN and ~0.2 μM dissolved inorganic phosphate, DIP), whereas treated nubbins were incubated in FSW enriched with 50 μM nitrate, a concentration selected to elicit clear responses while remaining within a range tolerated by corals grown under laboratory conditions. Coral nubbins were sampled in triplicate for each species at 0 h (prior to incubation in the experimental treatments), and after 3 and 6 h of incubation. Samples were immediately placed in liquid nitrogen for preservation at −80°C until downstream processing for WB.

For Experiment B, 18 N-depleted nubbins per coral species were sampled. Symbionts were freshly isolated from the coral host tissues (details in Supporting Information S11). Isolated symbionts from three nubbins of each species were pooled to constitute biological replicates (*n* = 3) of each treatment. Control FIS were incubated for 6 h in 70 ml of natural oligotrophic FSW, whereas treated FIS were incubated in 70 ml of natural FSW supplemented with 50 μM NO_3_^−^. After 3 and 6 h of incubation, 10 ml of FIS culture were sampled and immediately processed for protein extraction.

All samples were processed for protein extraction, quantification, and WB as described above for cultured Symbiodiniaceae strains. FIS samples were preserved in RNAlater solution (Thermo Scientific, USA) until downstream analysis for ITS2 rRNA gene sequencing (Supporting Information S1).

### Statistical analyses

Statistical analyses were performed in R (R version 4.3.1) [54]. Data were checked for normality and homoscedasticity. If normality and/or homoscedasticity conditions were not met, data were log-transformed (using base 10 logarithms). A two-way (time × treatment) repeated measure ANOVA (rmANOVA) was applied to analyse the responses in Experiments 1 and 2, as well as the experiments investigating NR expression in *in hospite* symbionts (Experiments A and B). To achieve this, linear mixed-effect (LME) models (lme() function, nlme R package, v3.1.163) were fitted to the data to investigate the response of NR protein and gene expression (dependent variable) to treatment (NO_3_^−^ and NH_4_^+^) and time (fixed effects, independent and interacting), simultaneously accounting for repeated measures on replicate algal cultures (random effect). Similarly, LME was used for Experiment 5 to investigate the effects of treatment (light, dark, and light + DCMU) and time on both NR protein and gene expression. One-way ANOVA was applied to the WB data from Experiment 4 to test the significance of treatment (varying [NO_3_^−^]/[NH_4_^+^] ratio) on NR protein expression. For Experiments 3 and 6, two-way ANOVAs were used to analyse the independent and interacting effects of nitrogen treatment and inhibitor treatments on NR protein expression. When significant effects were observed, *post hoc* Tukey HSD tests were performed. Differences were considered significant when *P* < .05.

## Results

### Effect of nitrogen source on NR expression

Our initial immunodetection assays revealed little to no detectable nitrate reductase signal in protein extracts from Symbiodiniaceae strains *B. minutum* SSB01 and *S. microadriaticum* RSStyP-A cultured under nitrogen-replete conditions, or from symbionts isolated from nitrogen-replete corals (Supporting Information S5). To enable robust and reproducible quantification of NR responses, all subsequent experiments in this study included a nitrogen depletion step prior to the various treatments. We first identified the individual effects of the nitrogen source on NR expression. Nitrogen-depleted *B. minutum* were incubated with 500 μM NO₃^−^ or NH₄^+^, and NR protein and gene expression were monitored at 0, 3, and 6 h. Both nitrogen source and time significantly influenced NR protein levels (Fig. 3A; 2-rmANOVA, *P* < .0001). NR protein signal, initially below the immunodetection threshold, was detectable at 3 h and increased further at 6 h with NO₃^−^ (Tukey HSD: *P* < .001). In contrast, NR remained below immunodetection limits under NH₄^+^. A complementary experiment using *S. microadriaticum* under the same experimental conditions (Fig. 3E) showed a significant increase in NR protein expression after 3 and 6 h of NO₃^−^ incubation compared to initial nitrogen-depleted conditions (Tukey HSD: *P* < .0001). By contrast, protein levels in algae exposed to NH₄^+^ did not change significantly (Tukey HSD: *P* > .5). To validate the identity of the immunoreactive band used for NR quantification, LC–MS/MS analysis was performed on the corresponding 100–120 kDa gel region from four independent *B. minutum* SSB01 cultures. Using Sequest HT, the NR candidate was identified with high confidence in both of two independent *B. minutum*-related protein databases, based on 42–43 distinct peptides, including 32–33 unique peptides and yielding 42–43% sequence coverage. The identified peptides mapped to corresponding regions of the NR candidate in both databases (Supporting Information S7). Consistently, preincubation of the antibody with the authentic manufacturer-supplied immunizing peptide strongly reduced recognition of both recombinant *A. thaliana* NR and the corresponding immunoreactive signal in *B. minutum* extracts (Supporting Information S8). Together, these independent approaches support assignment of the 100–120 kDa immunoreactive band to NR.

**Figure 3:**
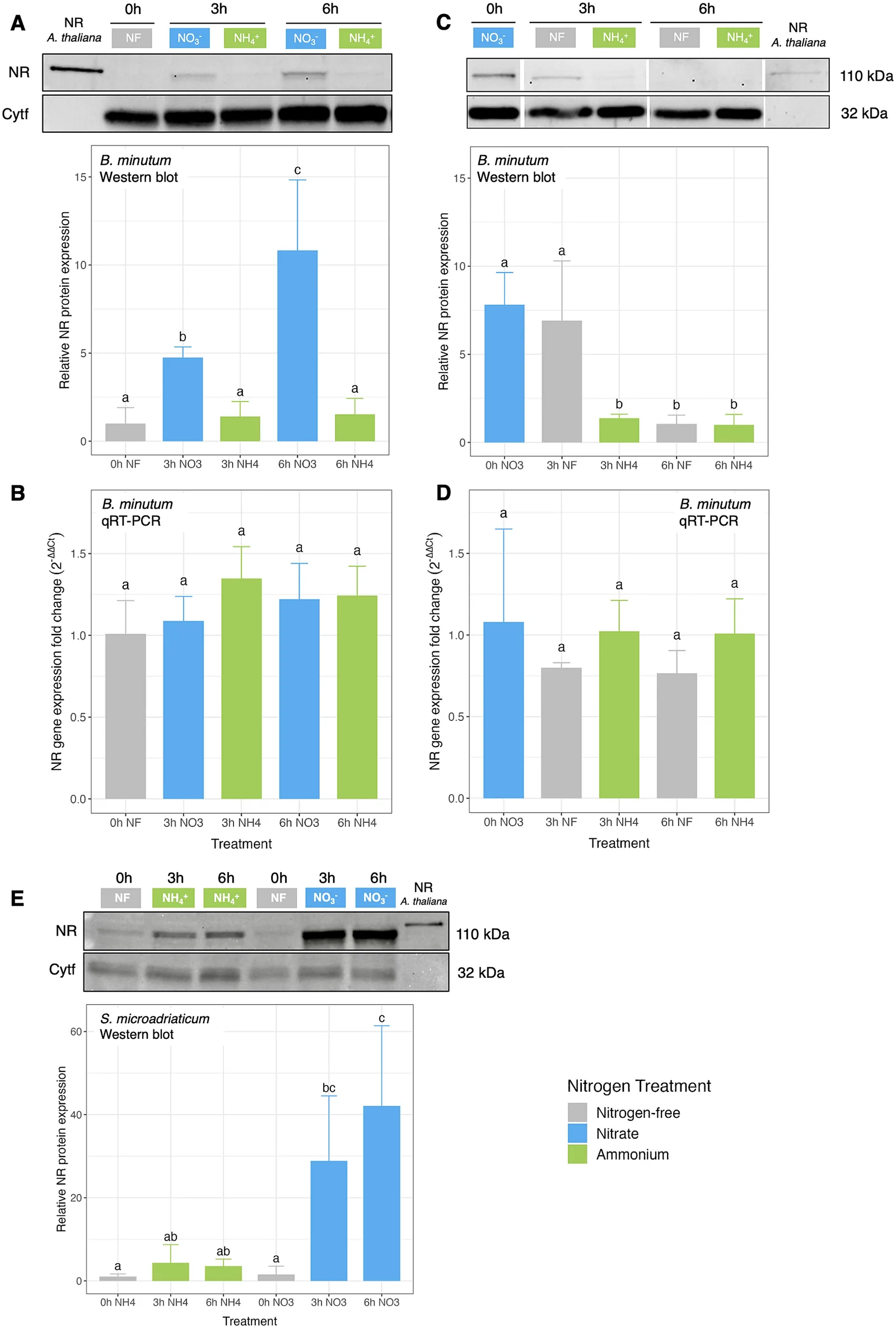
Kinetics of nitrate reductase expression in cultured Symbiodiniaceae. Experiment 1: NR protein (A) and gene (C) expression in *B. minutum,* and NR protein expression in *S. microadriaticum* (E) after incubation of N-depleted algae in f/2 medium supplemented with 500 μM NO_3_^−^ or 500 μM NH_4_^+^ for 3 and 6 h. Experiment 2: NR protein (B) and gene (D) expression in *B. minutum* following transfer of NR-expressing cells (preincubated 24 h in 500 μM NO_3_^−^) to NF or 500 μM NH_4_^+^ for 3 and 6 h. Each WB shows a representative membrane (out of three biological replicates), with NR protein (~110 kDa) and the loading control Cytf (~32 kDa). Mean relative WB signals intensities from all replicates are shown in the bar plots below. RT-qPCR results are represented as fold changes in *NR* gene expression (2^−∆∆Ct^) relative to time 0. Error bars represent 95% confidence intervals (CI) and statistically significant differences are indicated by different letters (Tukey HSD, *P* < .05).

NR protein degradation was next assessed in SSB01. NR-expressing cells were incubated in NF or NH₄^+^ media. Both time and treatment significantly affected NR expression (Fig. 3B; 2-rmANOVA, *P* < .01). After 3 h, NR remained stable in NF but decreased significantly with NH₄^+^, and after 6 h, NR fell below immunodetection limits in both conditions (Tukey HSD, *P* < .001). In both experiments, *NR* gene expressions in SSB01 remained unchanged regardless of nitrogen treatment or incubation time (Fig. 3C and D).

In Experiment 3, we explored how varying NO₃^−^/NH₄^+^ ratios affected NR expression. Algae were incubated for 6 h with 500 μM total nitrogen using NO₃^−^/NH₄^+^ ratios of 100–0, 98–2, 90–10, 75–25, 50–50, 25–75, and 0–100%. NR protein abundance declined significantly with decreasing NO₃^−^/NH₄^+^ ratios (ANOVA, *P* < .0001), though the response was nonlinear (Fig. 4). For example, 25% NH₄^+^ reduced NR expression to 60 ± 5% of the 100% NO₃^−^ value, whereas 50% NH₄^+^ resulted in 28 ± 9%, similar to levels in NF (16 ± 7%) and 100% NH₄^+^ (24 ± 12%) conditions (Tukey HSD, *P* > .05).

**Figure 4:**
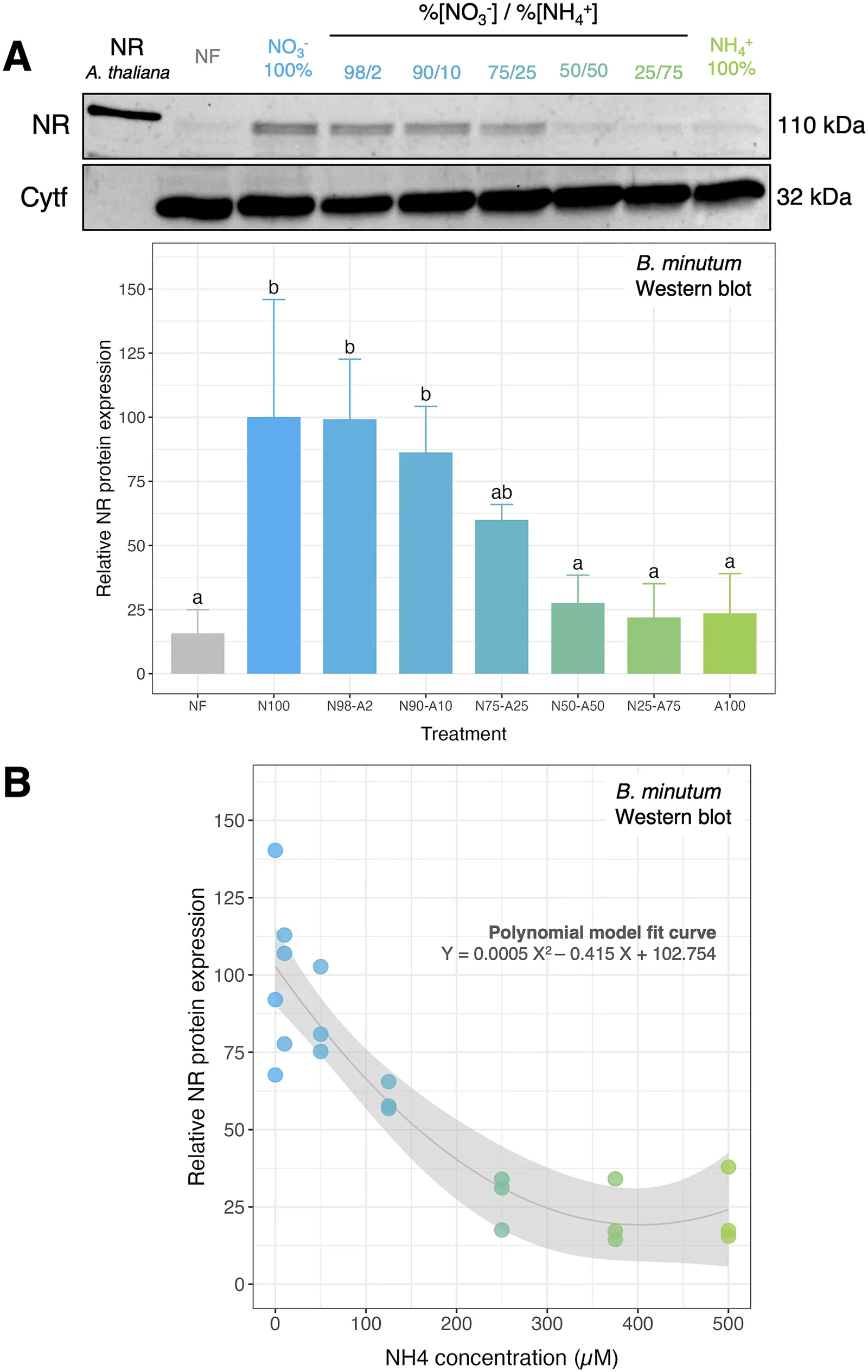
Effect of [NO_3_^−^]/[NH_4_^+^] concentration ratios on nitrate reductase expression in *B. minutum* SSB01. N-depleted SSB01 were incubated for 6 h in f/2 media containing 500 μM total nitrogen with varying NO_3_^−^/NH_4_^+^ concentrations ratios. (A) Representative WB membrane (out of three replicates) is shown. The upper band (~110 kDa) corresponds to the NR protein; the lower band (~32 kDa) to the loading control (Cytf). Mean relative WB signal intensities (*n* = 3) are shown in the bar plot below. Statistically significant differences are indicated by different letters. Error bars correspond to the 95% CI. (B) Scatter plot of relative NR expression as a function of NH_4_^+^ concentration, with a fitted polynomial regression (grey line) and 95% CI (grey band).

### Effect of light on NR expression

The influence of light on NR expression was next investigated. Nitrogen-depleted SSB01, dark-acclimated for 12 h, were incubated with 500 μM NO₃^−^ for 6 and 24 h under three light conditions: light (150 μmol photons m^−2^ s^−1^), light with 40 μM 3-(3,4-dichlorophenyl)-1,1-dimethylurea (DCMU) (an inhibitor of photosystem II (PSII) electron flow; [55, 56]), and complete darkness. Both light treatment and incubation time significantly affected NR protein expression (Fig. 5A; two-way ANOVA, *P* < .0001). As in previous experiments, NR protein abundance increased significantly after 6 h in light (Tukey HSD, *P* < .0001). The protein was also synthetized in darkness (*P* < .05), although at lower levels than in light (*P* < .0001) or light + DCMU (*P* = .01). After 24 h, NR protein abundance in darkness decreased to levels comparable to nitrogen-depleted cells and remained significantly lower under light + DCMU than under light (*P* = .0001).

**Figure 5:**
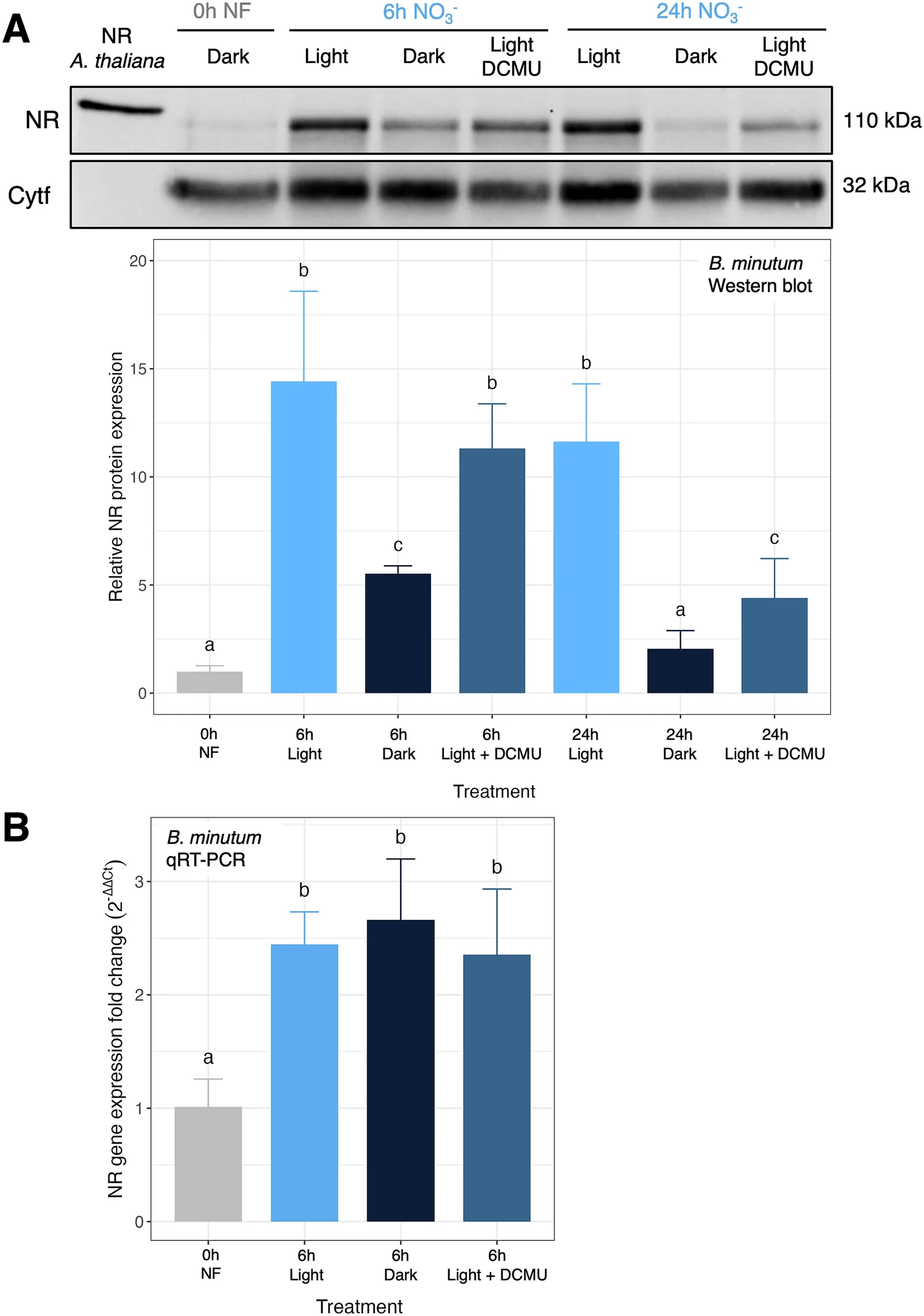
Role of light and photosynthesis in nitrate reductase expression in *B. minutum* SSB01. N-depleted SSB01 were incubated for 6 and 24 h in f/2 medium with 500 μM NO_3_^−^ under three light conditions (light, dark, and light + 40 μM DCMU). (A) Representative WB membrane (out of three replicates), the upper band (~110 kDa) corresponds to the NR protein; the lower band (~32 kDa) to the loading control (Cytf). Mean relative band intensities from all replicates (*n* = 3) are shown in the bar plot. (B) RT-qPCR results show *NR* gene expression fold change (2^−∆∆Ct^) relative to time 0. Error bars indicate 95% CI. Different letters indicate significant differences (Tukey HSD, *P* < .05).

*NR* gene expression, measured after 6 h of NO₃^−^ incubation, was significantly higher in NO₃^−^-treated cells than in nitrogen-depleted controls (ANOVA, *P* < .0001) but was not affected by the light treatments (Fig. 5B).

### Effect of transcription and translation inhibitors on NR expression

Because NR protein abundance did not correlate with *NR* gene expression in Experiments 1, 2, and 4, Experiment 5 examined potential transcriptional and post-transcriptional regulation using specific inhibitors of transcription (ACT-D and CDC) and translation (CHX). ACT-D binds DNA and CDC disrupts mRNA polyadenylation [57, 58], whereas CHX blocks protein elongation [59]. Both NF and NO₃^−^ conditions were used, with the former serving as a control due to naturally lower NR protein levels.

Nitrogen level and inhibitor treatment each had a significant effect on NR protein expression (two-way ANOVA: *P* < .01), with no interaction effect, indicating consistent responses across conditions (Fig. 6A). NR protein remained low in NF and NF + ACT-D/CDC, similar to the nitrogen-depleted and NF + CHX treatments (Tukey HSD, *P* > .05). In contrast, NR protein expression increased significantly in NO₃^−^ and NO₃^−^ + ACT-D/CDC (*P* > .01) but was suppressed in NO₃^−^ + CHX, to levels indistinguishable from NF (*P* > .05). *NR* gene expression was not affected by nitrogen or inhibitor treatments (two-way ANOVA, *P* > .05 for all effects) (Fig. 6B).

**Figure 6:**
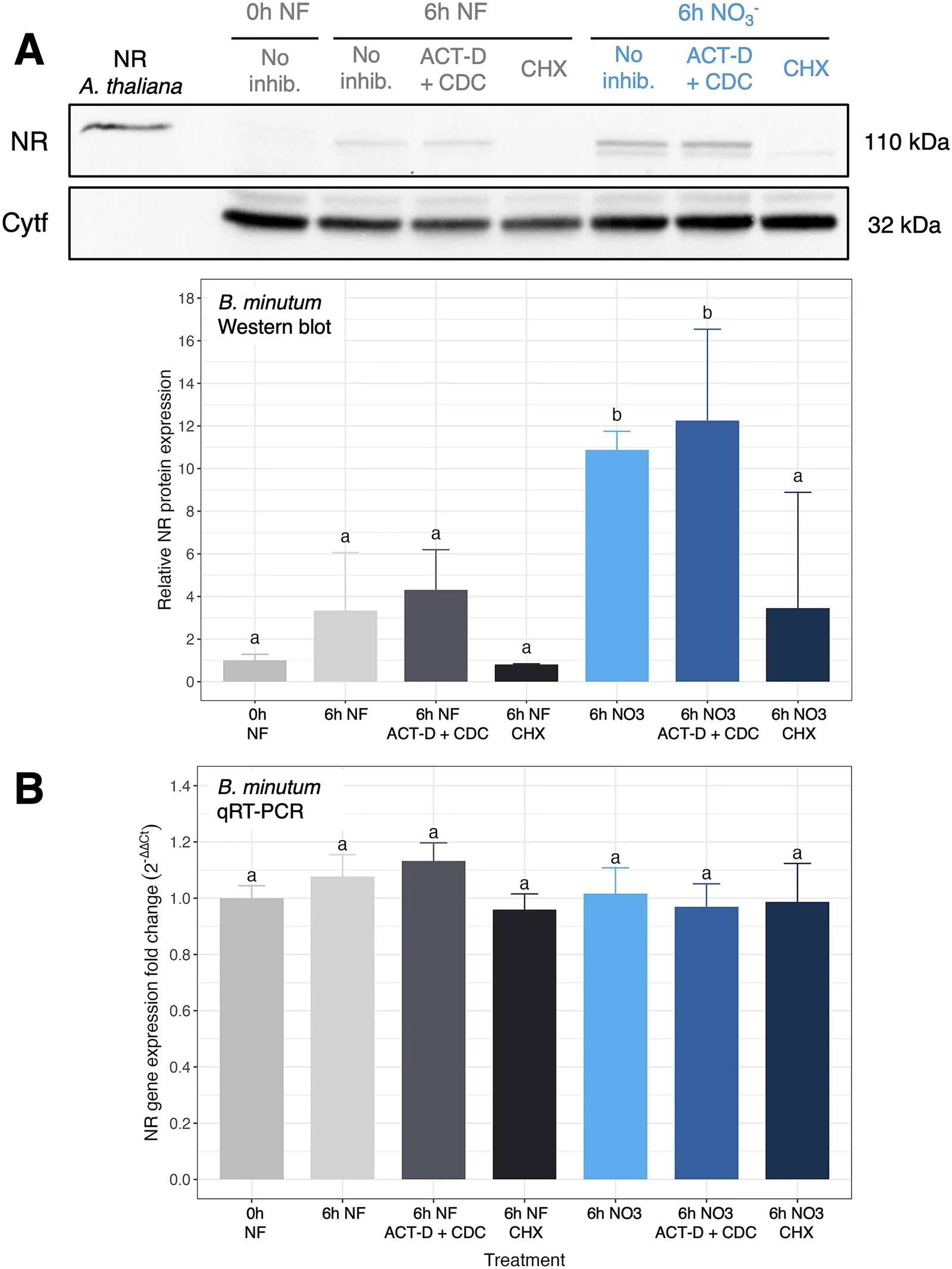
Effect of transcription and translation inhibitors on nitrate reductase expression in *B. minutum* SSB01. N-depleted SSB01 were incubated in NF f/2 NF or 500 μM NO_3_^−^, with or without transcription inhibitors (ACT-D + CDC), or translation inhibitor CHX. (A) Representative WB membrane (out of three biological replicates), the upper band (~110 kDa) corresponds to the NR protein; the lower band (~32 kDa) to the loading control (Cytf). Bar plot of the mean relative WB signal from all replicates (*n* = 3). (B) RT-qPCR results expressed as *NR* gene fold change (2^−∆∆Ct^) relative to time 0. Error bars indicate 95% CI. Different letters denote significant differences (Tukey HSD, *P* < .05).

### Expression of NR in coral symbionts

Building on previous experiments in cultured Symbiodiniaceae strains under NO₃^−^-enriched conditions, we next examined NR protein expression in symbiotic contexts. Experiments A and B assessed NR expression in *in hospite* (coral tissue) and freshly isolated natural symbionts (FIS), respectively, using three coral species with known ITS2 symbiont profiles. *S. pistillata* predominantly hosted type A1 Symbiodiniaceae (*S. microadriaticum*, 98%), whereas *T. reniformis* and *E. lamellosa* mainly harboured *Cladocopium* (86% and 64%), with *E. lamellosa* also containing *Durusdinium* (25%).

Preliminary tests failed to detect NR protein in *S. pistillata* symbionts under nitrate-enriched conditions without prior nitrogen depletion (Supporting Information S12). Thus, in subsequent incubations, corals were first nitrogen-starved, then exposed to either natural FSW or FSW enriched with 50 μM NO₃^−^, following protocols shown to induce NR expression in cultured Symbiodiniaceae strains.

NR protein was detected *in hospite* by WB in all three species after 3 h of incubation in both control and NO₃^−^-enriched seawater (Fig. 7A,C, and  E). Expression tended to be higher under NO₃^−^ enrichment but only reached statistical significance in *E. lamellosa* (Tukey HSD, *P* = .016), likely due to high variability and low detection levels in *S. pistillata* and *T. reniformis*. In all cases, NR levels were significantly lower after 6 h compared to the 3 h NO₃^−^ condition (*P* < .05).

**Figure 7:**
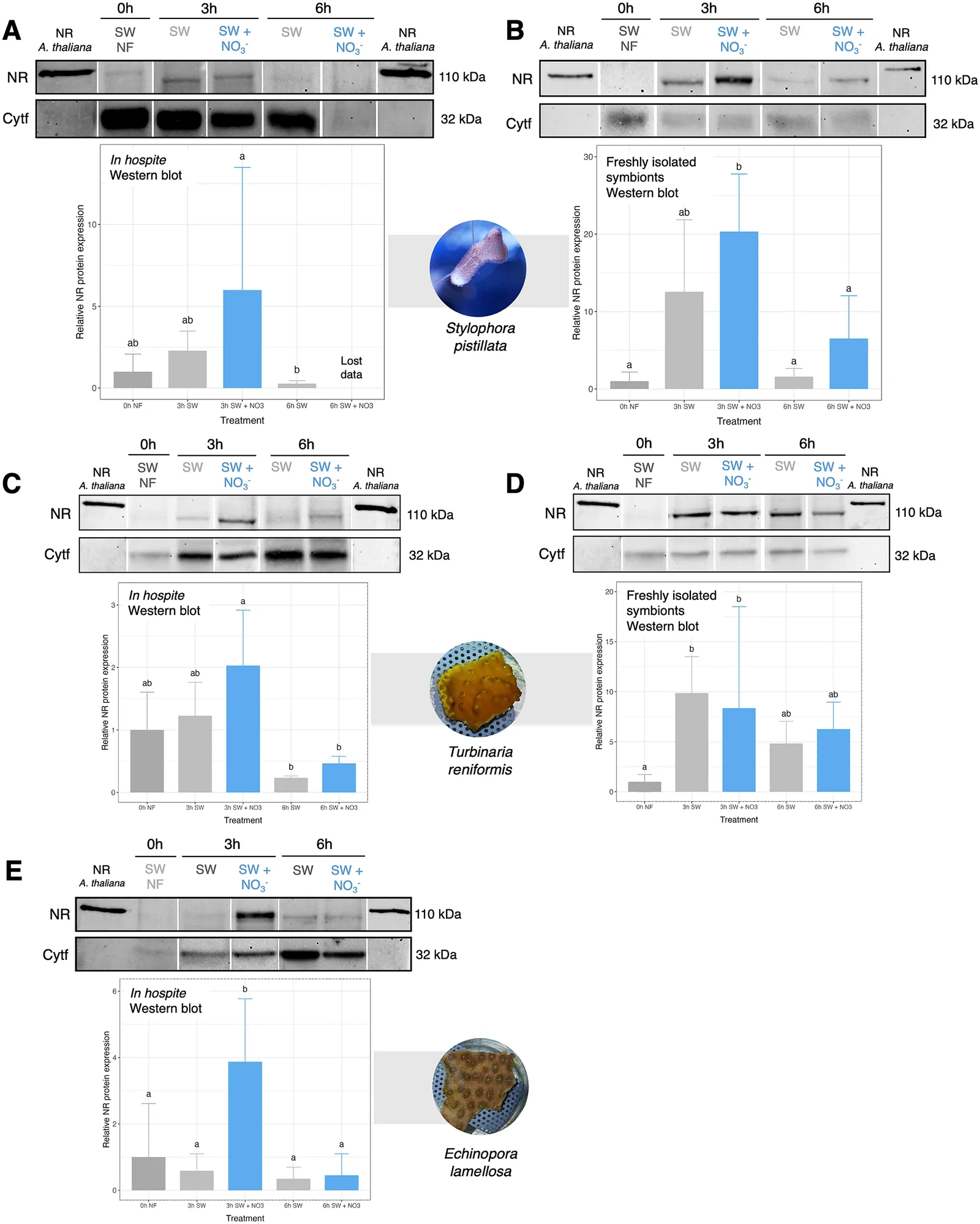
Nitrate reductase protein expression in symbionts from three coral species, *in hospite* and after isolation. WB analysis of NR protein expression in symbionts from N-depleted corals, *S. pistillata* (A), *T. reniformis* (C), and *E. lamellosa* (E), incubated for 3 and 6 h in either natural seawater (SW, ~0.5–0.7 μM DIN) or natural SW enriched with 50 μM NO_3_^−^. NR expression in FIS from *S. pistillata* (B) and *T. reniformis* (D) under the same incubation conditions. For clarity, only one representative WB band per condition (from a total of three biological replicates) is shown. The upper band (~110 kDa) corresponds to the NR protein; the lower band (~32 kDa) to the loading control (Cytf). Recombinant *A. thaliana* NR (Sigma-Aldrich N0163; expected and apparent molecular weights of ~103 and 117 kDa, respectively) was used as a positive control. Bar plots below each blot show mean relative NR signal intensities (*n* = 3). Error bars represent the 95% CI, and different letters indicate statistically significant differences (Tukey HSD, *P* < .05).

In FIS, NR protein was detected under both FSW and NO₃^−^-enriched conditions after 3 and 6 h in symbionts from *S. pistillata* and *T. reniformis* (Fig. 7B and D). Although differences were not statistically significant due to high variability (*P* > .5), NR expression in *S. pistillata* FIS was consistently higher under NO₃^−^, a pattern not observed in *T. reniformis*.

## Discussion

Nitrate is the most abundant form of DIN in coral reef environments but exhibits strong temporal and spatial variability due to both natural processes and anthropogenic inputs [7, 60, 61]. Understanding how nitrate is assimilated by the coral holobiont is therefore crucial for elucidating nitrogen cycling and coral resilience. Since nitrate reductase activity was first reported in natural Symbiodiniaceae [15], little attention has been given to NR protein expression itself. Most subsequent studies have inferred nitrate assimilation from uptake measurements at the holobiont level, generally assuming that symbiont NR activity underpins this process [9, 10, 16, 62]. Here, we provide direct evidence of NR protein and gene expression in Symbiodiniaceae exposed to different nitrogen sources, revealing that NR is regulated at multiple levels.

### Regulation of NR by nitrogen source and NH₄^+^ inhibition

Our results demonstrate that NR is substrate-induced: nitrate triggers its synthesis in nitrogen-starved Symbiodiniaceae strains, leading to significant NR protein accumulation, whereas ammonium does not (Fig. 3A,B, and  E). This pattern, observed in *B. minutum* and supported by complementary results in *S. microadriaticum*, parallels findings in higher plants, chlorophytes, and diatoms, where NR expression is tightly regulated by nitrate availability and repressed by more reduced nitrogen forms [63]. However, although the complementary results in *S. microadriaticum* support a consistent nitrate-induced NR accumulation pattern, additional coordinated experiments across multiple Symbiodiniaceae species are required to assess the broader taxonomic relevance of this response. We further observed that in the presence of NH₄^+^, NR protein in *B. minutum* declines more rapidly (within 3 h) than under nitrogen starvation alone (Fig. 3B), indicating that ammonium exerts a direct inhibitory effect on protein expression. Previous studies have reported lower NO_3_^−^ uptake rates by Symbiodiniaceae [9, 23, 64] and coral holobionts [10, 65], as well as the downregulation of most NRT genes in the presence of NH_4_^+^ [23]. By combining these previous observations with our results, we deduce that ammonium acts as a potent suppressor of the nitrate assimilation pathway, acting at both the level of transport and NR protein availability.

NR synthesis in cultured *B. minutum* is further modulated by the relative availability of nitrate and ammonium. When supplied together, NR expression declined sharply beyond a threshold ratio of NH_4_^+^ to NO_3_^−^ (Fig. 4), suggesting a switch-like response. Equimolar concentrations were sufficient to fully repress NR down to levels measured in NF conditions, consistent with earlier findings that NO_3_^−^ uptake ceases when NH_4_^+^ constitutes >25% of total nitrogen [64]. These results indicate that ammonium exerts a concentration-dependent, active repression of NR expression, reflecting a hierarchy in nitrogen source preference.

### Post-transcriptional and post-translational regulation

Whether ammonium represses NR synthesis or promotes its degradation remains unclear. In chlorophyte algae, both processes have been implicated [66], but regulatory pathways in dinoflagellates are still poorly understood. Given their unusual genomic organization-tandem gene arrays, permanently condensed chromosomes, and a paucity of canonical transcription factors [67–70], Symbiodiniaceae are thought to rely heavily on post-transcriptional control. Consistent with this, *NR* mRNA levels in SSB01 remained stable across treatments (Fig. 3), supporting the view that NR regulation occurs primarily at the post-transcriptional or post-translational levels. A similar lack of coordinated transcriptional response between nitrate and ammonium treatments was reported in the Symbiodiniaceae *Fugacium kawagutii,* where several *NR*-annotated transcripts showed heterogeneous expression patterns [23]. Because extensive gene duplication and the absence of well-resolved reference sequences complicate isoform-specific analyses in Symbiodiniaceae, transcriptomic results must be interpreted with caution. In particular, individual *NR*-annotated transcripts may represent distinct gene copies with different regulatory dynamics. The use of primers targeting a conserved catalytic region is therefore expected to capture the pool of functional *NR* transcripts, providing a complementary perspective when integrated with protein-level analyses.

Pharmacological inhibition experiments further support predominant post-transcriptional regulation of NR in *B. minutum*. NR protein levels declined with CHX, which blocks translation [59], but remained unaffected by the transcriptional inhibitors ACT-D and CDC [57, 58] (Fig. 6). This pattern indicates that NR protein accumulation depends primarily on ongoing translation rather than on changes in transcript abundance. This is consistent with emerging evidence of pervasive post-transcriptional mechanisms in dinoflagellates, including small RNA-mediated regulation and RNA editing via pentatricopeptide repeat (PPR) proteins [38, 71–74]. The constitutive stability of *NR* transcripts likely enables rapid enzyme synthesis when nitrate becomes available, which could constitute an adaptive trait for organisms experiencing fluctuating nitrogen regimes.

Post-translational regulation may provide an additional control layer. In higher plants, NR activity is modulated via phosphorylation-dependent binding of 14-3-3 proteins, leading to reversible inactivation [75], or to irreversible inhibition by protein binding, and targeted proteolysis [76]. Although such mechanisms have not yet been described in dinoflagellates, similar processes could fine-tune NR activity in response to environmental cues.

### Light-dependent regulation

Nitrate assimilation is energetically linked to photosynthesis, as NR requires photosynthetically derived electrons to reduce nitrate [77]. Whereas light-dependent NR activity is well established in plants and algae [63, 78–80], evidence in Symbiodiniaceae strains and coral holobionts has been inconsistent [9, 10, 15, 16, 22]. Our results show that NR protein levels decline in darkness and are partially suppressed by DCMU, implicating PSII-derived electron flow in maintaining NR protein abundance (Fig. 5A). Similar effects of DCMU on NR activity and NO₃^−^ assimilation have been reported in various photoautotrophs [79–83]. However, alternative regulatory mechanisms have also been proposed, including regulation via PSI, the redox state of the plastoquinone pool, or metabolic signals such as sugars and their derivatives [79, 84–86]. Taken together, these observations suggest that NR synthesis in Symbiodiniaceae depends on nitrate availability, whereas its stability and activation may depend on photosynthetic electron transport and cellular redox state.

*NR* gene expression patterns differed among experiments depending on sampling under light or dark conditions (Fig. 3B and D vs. Fig. 5A). This likely reflects light-responsive transcription, consistent with reports that dinoflagellate gene expression can vary diurnally even in the absence of strong circadian control [34, 43, 87, 88]. *NR* transcription may therefore follow a binary “on–off” mode triggered by light or nitrate exposure, whereas post-transcriptional mechanisms determine protein abundance.

### NR expression in symbiotic context

Although our culture experiments clarify NR regulation in free-living *B. minutum* and confirm the role of nitrate in inducing NR protein synthesis across both *B. minutum* and *S. microadriaticum* strains, symbionts function within the highly controlled environment of the coral host [89]. We demonstrate that natural symbionts within three coral species (*S. pistillata*, *T. reniformis*, and *E. lamellosa*) can accumulate NR protein *in hospite* following nitrate exposure, providing evidence that NR protein can be induced in symbiosis (Fig. 7A,C, and  E). These observations suggest that exogenous NO_3_^−^ was transported through host tissues to the symbionts, where NR protein accumulation was detectable within 3 h. The absence of NH_4_^+^-mediated repression *in hospite* suggests that intracellular NH_4_^+^ concentrations were insufficient to inhibit NR protein synthesis under nitrogen-depleted conditions. After 6 h, NR protein abundance declined across all coral species, suggesting that NR protein accumulation represents a transient response to newly available nitrate under nitrogen-depleted conditions.

Measurements in FIS further revealed that NR protein in *S. pistillata* symbionts increased in a nitrate concentration-dependent manner (Fig. 7B), consistent with patterns observed in cultured *S. microadriaticum* (Fig. 3E). In contrast, FIS from *T. reniformis* showed no difference between natural and NO_3_^−^-enriched conditions (Fig. 7D). The natural seawater used in these experiments contains low levels of NO_3_^−^ (DIN 0.5–0.7 μM), which may be sufficient to induce NR synthesis. NR protein may also be expressed constitutively at low levels, with synthesis increasing in response to elevated NO_3_^−^ concentrations. Additionally, species-specific nitrogen acquisition strategies [90, 91] could differentially influence the regulation of nitrogen assimilation enzymes. In FIS, NR protein signals normalized to Cytf and NR-positive controls were substantially higher than those measured *in hospite*. Although WB is semiquantitative, this consistent pattern suggests that NR protein abundance is lower in symbionts residing within the host environment. Such repression likely reflects both reduced nitrate availability, due to dependence on host-mediated transport across host and symbiosome membranes, and the presence of alternative nitrogen sources supplied by the host, including NH₄^+^, urea, and amino acids.

Strategies of host-symbiont nitrogen exchange differ among coral species and symbiont lineages. *S. pistillata*, for instance, has been shown to rely more strongly on direct transfer of heterotrophically derived amino acids to its symbionts, whereas in *T. reniformis*, heterotrophic nitrogen appears to be primarily routed through NH_4_^+^ recycling [92]. Corals harbouring *Cladocopium* typically exhibit higher heterotrophic capacity but lower inorganic nitrogen assimilation than those hosting *Symbiodinium* [93], although *Cladocopium* has been shown to assimilate NO_3_^−^ more efficiently than *Durusdinium* [90]. NR expression in symbiotic Symbiodiniaceae is therefore likely to be finely tuned to the coral’s nutritional state and to the relative availability of organic and inorganic nitrogen sources. When organic nitrogen or ammonium is readily available, NR expression appears to be reduced or suppressed, consistent with our observation that NR protein was undetectable in *S. pistillata* symbionts under regular feeding conditions without nitrogen depletion (Supporting Information S12).

The ammonium-dependent regulation of NR has important ecological implications for nitrogen cycling within cnidarian-algal symbioses. Variability in nitrate assimilation across symbiotic cnidarians likely reflects differences in trophic strategy and nitrogen availability. Highly heterotrophic taxa, such as *Cassiopea* spp., often show limited or undetectable nitrate assimilation, likely reflecting reduced reliance on symbiont-driven nitrogen acquisition when particulate feeding supplies sufficient reduced nitrogen [94, 95]. Similarly, several reef-building corals and anemones exhibit reduced nitrate uptake under aquarium conditions, where ammonium availability is typically elevated [10, 20]. Such regulatory feedback likely confers several adaptive advantages. Although nitrate represents an additional nitrogen source in oligotrophic reef waters, its assimilation primarily benefits the symbionts, as assimilated nitrate appears to be largely retained within endosymbiont cells, with limited transfer to host tissues [89, 96]. Consequently, unchecked nitrate assimilation could favour symbiont proliferation and weaken the nitrogen limitation that contributes to the stability of the symbiosis [89, 97]. Ammonium-mediated repression of NR may therefore contribute to maintaining host control over symbiont nitrogen acquisition while minimizing the energetic costs of nitrate reduction, an ATP- and reductant-intensive process [11]. Such regulation would allow the holobiont to exploit transient nitrate pulses while preferentially assimilating ammonium, thereby optimizing nitrogen-use efficiency in oligotrophic environments. However, this strategy may also entail costs, as repression of nitrate assimilation under chronically ammonium-enriched or heterotrophic conditions could reduce metabolic flexibility in fluctuating nutrient regimes, potentially constraining nitrate utilization when reduced nitrogen supply declines.

The experimental results presented here should be interpreted in light of several methodological considerations inherent to protein-based analyses. Although a key finding of this study is that nitrogen depletion, either in free-living cells or within the coral host, is required for detectable NR induction in response to nitrate enrichment, protein immunodetection has finite sensitivity and does not directly measure enzymatic activity. Accordingly, low or undetectable NR protein levels do not necessarily imply the absence of nitrate reduction, as small basal NR pools combined with strong post-translational regulation may be sufficient to sustain nitrate assimilation. Conversely, immunodetected NR proteins do not necessarily represent catalytically active enzymes, as inactive (i.e. phosphorylated) forms can also be recognized by the antibodies. In addition, because nitrate reductase proteins in Symbiodiniaceae have not yet been biochemically characterized, WB analyses necessarily reflect the accumulation of NR-immunoreactive proteins rather than the unequivocal detection of a specific, functionally validated isoform. The relatively high nutrient concentrations used in this study, although not fully representative of natural reef conditions, were intentionally selected to elicit clear and reproducible responses in laboratory-maintained Symbiodiniaceae and corals, which are typically acclimated to elevated nutrient levels. Despite these constraints, our findings advance a protein-level perspective on NR regulation in Symbiodiniaceae and establish a foundation for future studies integrating enzyme activity measurements and ecologically relevant nutrient regimes across multiple Symbiodiniaceae and coral species.

## Conclusion

The results presented here collectively indicate that Symbiodiniaceae exhibit a tightly regulated, substrate-induced NR system that enables rapid yet transient NR induction under nitrogen-depleted conditions. NR protein accumulation is detectable within hours of nitrate availability and is actively suppressed by ammonium, likely through predominantly post-transcriptional mechanisms. This rapid regulation may reflect an adaptive strategy to fluctuating nutrient environments, allowing symbionts to exploit transient nitrate pulses without compromising nitrogen homeostasis. Within the coral holobiont, these findings suggest the potential for an auxiliary nitrogen-acquisition pathway that complements ammonium recycling and heterotrophic nitrogen transfer.

More broadly, these findings highlight the importance of post-transcriptional and post-translational control in Symbiodiniaceae nitrogen metabolism and underscore the limitations of relying exclusively on transcriptomic data to infer functional responses. Future studies integrating proteomic, metabolomic, and enzyme activity measurements [98–102] will be essential to fully elucidate nitrogen regulation within the coral symbiosis and its implications for holobiont nutrient balance and resilience.

## Supplementary Material

Supplementary_Material_wrag260

## Data Availability

Data are available via the Zenodo repository: https://doi.org/10.5281/zenodo.18352345. The mass spectrometry proteomics data have been deposited to the ProteomeXchange Consortium via the PRIDE [103] partner repository with the dataset identifier PXD083567 and 10.6019/PXD083567.

## References

[1] Stévenne C, Micha M, Plumier JC, et al. Corals and sponges under the light of the holobiont concept: how microbiomes underpin our understanding of marine ecosystems. *Front Mar Sci* 2021;8:698853. 10.3389/fmars.2021.698853

[2] Yellowlees D, Rees TAV, Leggat W. Metabolic interactions between algal symbionts and invertebrate hosts. *Plant Cell Environ* 2008;31:679–94. 10.1111/j.1365-3040.2008.01802.x 18315536

[3] Muller-Parker G, D’Elia CF, Cook CB. Interactions between corals and their symbiotic algae. In: Birkeland C. (ed.), Coral Reefs in the Anthropocene. Dordrecht: Springer, 2015, 99–116 10.1007/978-94-017-7249-5_5.

[4] Houlbrèque F, Ferrier-Pagès C. Heterotrophy in tropical scleractinian corals. *Biol Rev* 2009;84:1–17. 10.1111/j.1469-185X.2008.00058.x 19046402

[5] Gustafsson MSM, Baird ME, Ralph PJ. The interchangeability of autotrophic and heterotrophic nitrogen sources in Scleractinian coral symbiotic relationships: a numerical study. *Ecol Model* 2013;250:183–94. 10.1016/J.ECOLMODEL.2012.11.003

[6] Conti-Jerpe IE, Thompson PD, Wong CWM, et al. Trophic strategy and bleaching resistance in reef-building corals. *Sci Adv* 2020;6:eaaz5443. 10.1126/sciadv.aaz5443 PMC7148090 32300659

[7] Gruber N . The marine nitrogen cycle: Overview and challenges. In: Capone D.G. et al. (eds.), Nitrogen in the Marine Environment. San Diego: Academic Press, 2008, 1–50 10.1016/B978-0-12-372522-6.00001-3.

[8] Dagenais-Bellefeuille S, Morse D. Putting the N in dinoflagellates. *Front Microbiol* 2013;4:369. 10.3389/fmicb.2013.00369 PMC3849724 24363653

[9] Domotor SL, D’Elia CF. Nutrient uptake kinetics and growth of zooxanthellae maintained in laboratory culture. *Mar Biol* 1984;80:93–101. 10.1007/BF00393132

[10] Grover R, Maguer JF, Allemand D, et al. Nitrate uptake in the scleractinian coral *Stylophora pistillata*. *Limnol Oceanogr* 2003;48:2266–74. 10.4319/lo.2003.48.6.2266

[11] Sanz-Luque E, Chamizo-Ampudia A, Llamas A, et al. Understanding nitrate assimilation and its regulation in microalgae. *Front Plant Sci* 2015;6:899. 10.3389/fpls.2015.00899 PMC4620153 26579149

[12] Rädecker N, Pogoreutz C, Voolstra CR, et al. Nitrogen cycling in corals: the key to understanding holobiont functioning? *Trends Microbiol* 2015;23:490–7. 10.1016/j.tim.2015.03.008 25868684

[13] Tang J, Cai W, Yan Z, et al. Involvement of the ammonium assimilation mediated by glutamate dehydrogenase in response to heat stress in the scleractinian coral *Pocillopora damicornis*. *J Oceanol Limnol* 2022;40:2001–11. 10.1007/s00343-021-1266-8

[14] Gibbin EM, Krueger T, Putnam HM, et al. Short-term thermal acclimation modifies the metabolic condition of the coral holobiont. *Front Mar Sci* 2018;5:10. 10.3389/fmars.2018.00010

[15] Crossland CJ, Barnes DJ. Nitrate assimilation enzymes from two hard corals, *Acropora acuminata* and *Goniastrea australensis*. *Comp Biochem Physiol Part B Comp Biochem* 1977;57:151–7. 10.1016/0305-0491(77)90165-1

[16] Badgley BD, Lipschultz F, Sebens KP. Nitrate uptake by the reef coral *Diploria strigosa*: effects of concentration, water flow, and irradiance. *Mar Biol* 2006;149:327–38. 10.1007/s00227-005-0180-5

[17] Tanaka Y, Grottoli AG, Matsui Y, et al. Partitioning of nitrogen sources to algal endosymbionts of corals with long-term 15N-labelling and a mixing model. *Ecol Model* 2015;309-310:163–9. 10.1016/j.ecolmodel.2015.04.017

[18] DiRoberts LE, Dudek A, Ray NE, et al. Testing assumptions of nitrogen cycling between a temperate, model coral host and its facultative symbiont: symbiotic contributions to dissolved inorganic nitrogen assimilation. *Mar Ecol Prog Ser* 2021;670:61–74. 10.3354/MEPS13731

[19] Wilkerson FP, Trench RK. Nitrate assimilation by zooxanthellae maintained in laboratory culture. *Mar Chem* 1985;16:385–93. 10.1016/0304-4203(85)90059-3

[20] Wilkerson FP, Muscatine L. Uptake and assimilation of dissolved inorganic nitrogen by a symbiotic sea anemone. *Proc R Soc Lond Ser B Biol Sci* 1984;221:71–86. 10.1098/rspb.1984.0023

[21] Miller DJ, Yellowlees D. Inorganic nitrogen uptake by symbiotic marine cnidarians: a critical review. *Proc R Soc B Biol Sci* 1989;237:109–25. 10.1098/rspb.1989.0040

[22] D’Elia CF, Domotor SL, Webb KL. Nutrient uptake kinetics of freshly isolated zooxanthellae. *Mar Biol* 1983;75:157–67. 10.1007/BF00405998

[23] Li T, Chen X, Lin S. Physiological and transcriptomic responses to N-deficiency and ammonium: nitrate shift in *Fugacium kawagutii* (Symbiodiniaceae). *Sci Total Environ* 2021;753:141906. 10.1016/j.scitotenv.2020.141906 32890873

[24] Xiang T, Lehnert E, Jinkerson RE, et al. Symbiont population control by host-symbiont metabolic interaction in Symbiodiniaceae-cnidarian associations. *Nat Commun* 2020;11:108. 10.1038/s41467-019-13963-z PMC6949306 31913264

[25] Kirk AL, Clowez S, Lin F, et al. Transcriptome reprogramming of Symbiodiniaceae *Breviolum minutum* in response to casein amino acids supplementation. *Front Physiol* 2020;11:574654. 10.3389/fphys.2020.574654 PMC7710908 33329024

[26] Aranda M, Li Y, Liew YJ, et al. Genomes of coral dinoflagellate symbionts highlight evolutionary adaptations conducive to a symbiotic lifestyle. *Sci Rep* 2016;6:39734. 10.1038/srep39734 PMC5177918 28004835

[27] Pang CZ, Ip YK, Chew SF. Using transcript levels of nitrate transporter 2 as molecular indicators to estimate the potentials of nitrate transport in *Symbiodinium*, *Cladocopium*, and *Durusdinium* of the fluted giant clam *Tridacna squamosa*. *Front Mar Sci* 2021;8:1821. 10.3389/fmars.2021.784662

[28] Sproles AE, Kirk NL, Kitchen SA, et al. Phylogenetic characterization of transporter proteins in the cnidarian-dinoflagellate symbiosis. *Mol Phylogenet Evol* 2018;120:307–20. 10.1016/j.ympev.2017.12.007 29233707

[29] Tortorelli G, Oakley CA, Davy SK, et al. Cell wall proteomic analysis of the cnidarian photosymbionts *Breviolum minutum* and *Cladocopium goreaui*. *J Eukaryot Microbiol* 2022;69:e12870. 10.1111/JEU.12870 PMC9293036 34448326

[30] Ip YK, Hiong KC, Teng JHQ, et al. The fluted giant clam (*Tridacna squamosa*) increases nitrate absorption and upregulates the expression of a homolog of SIALIN (H+:2NO3− cotransporter) in the ctenidium during light exposure. *Coral Reefs* 2020;39:451–65. 10.1007/s00338-020-01907-9

[31] Xiang T, Hambleton EA, DeNofrio JC, et al. Isolation of clonal axenic strains of the symbiotic dinoflagellate *Symbiodinium* and their growth and host specificity. *J Phycol* 2013;49:447–58. 10.1111/jpy.12055 27007034

[32] RRL G, Ryther JH. Studies of marine planktonic diatoms I. Cyclotella nana Hustedt and Detonula confervacea (Cleve) Gran. *Can J Microbiol* 1962;8:229–39. 10.1139/m62-029 13902807

[33] Guillard RRL . Culture of phytoplankton for feeding marine invertebrates. In: Smith W.L., Chanley M.H. (eds.), Culture of Marine Invertebrate Animals. Boston: Springer, 1975, 26–60 10.1007/978-1-4615-8714-9_3.

[34] Roy S, Beauchemin M, Dagenais-Bellefeuille S, et al. The Lingulodinium circadian system lacks rhythmic changes in transcript abundance. *BMC Biol* 2014;12:107. 10.1186/s12915-014-0107-z PMC4298066 25526979

[35] Bajhaiya AK, Dean AP, Zeef LAH, et al. PSR1 is a global transcriptional regulator of phosphorus deficiency responses and carbon storage metabolism in *Chlamydomonas reinhardtii*. *Plant Physiol* 2016;170:1216–34. 10.1104/pp.15.01907 PMC4775146 26704642

[36] Voigt J, Deinert B, Bohley P. Subcellular localization and light-dark control of ornithine decarboxylase in the unicellular green alga *Chlamydomonas reinhardtii*. *Physiol Plant* 2000;108:353–60. 10.1034/J.1399-3054.2000.T01-1-100403.X

[37] Laemmli UK . Cleavage of structural proteins during the assembly of the head of bacteriophage T4. *Nature* 1970;227:680–5. 10.1038/227680A0 5432063

[38] Shoguchi E, Shinzato C, Kawashima T, et al. Draft assembly of the *Symbiodinium minutum* nuclear genome reveals dinoflagellate gene structure. *Curr Biol* 2013;23:1399–408. 10.1016/j.cub.2013.05.062 23850284

[39] Mashini AG, Oakley CA, Beepat SS, et al. The influence of symbiosis on the proteome of the Exaiptasia endosymbiont *Breviolum minutum*. *Microorganisms* 2023;11:292. 10.3390/microorganisms11020292 PMC9967746 36838257

[40] Mashini A, Oakley CA, Peng L, et al. Proteomes of native and non-native symbionts reveal responses underpinning host-symbiont specificity in the cnidarian-dinoflagellate symbiosis. *ISME J* 2024;18:122. 10.1093/ismejo/wrae122 PMC11473927 38988135

[41] Parkinson JE, Baumgarten S, Michell CT, et al. Gene expression variation resolves species and individual strains among coral-associated dinoflagellates within the genus *Symbiodinium*. *Genome Biol Evol* 2016;8:665–80. 10.1093/gbe/evw019 PMC4824173 26868597

[42] Maruyama S, Unsworth JR, Sawiccy V, et al. Algae from *Aiptasia* egesta are robust representations of Symbiodiniaceae in the free-living state. *PeerJ* 2022;10:e13796. 10.7717/PEERJ.13796 PMC9341449 35923894

[43] Mayfield AB, Hsiao YY, Chen HK, et al. Rubisco expression in the dinoflagellate *Symbiodinium* sp. is influenced by both photoperiod and endosymbiotic lifestyle. *Mar Biotechnol* 2014;16:371–84. 10.1007/s10126-014-9558-z 24449387

[44] Scucchia F, Nativ H, Neder M, et al. Physiological characteristics of *Stylophora pistillata* larvae across a depth gradient. *Front Mar Sci* 2020;7:13. 10.3389/fmars.2020.00013 PMC6986922 31993434

[45] Winters G, Beer S, Zvi BB, et al. Spatial and temporal photoacclimation of *Stylophora pistillata*: zooxanthella size, pigmentation, location and clade. *Mar Ecol Prog Ser* 2009;384:107–19. 10.3354/MEPS08036

[46] Mieog JC, van Oppen MJH, Cantin NE, et al. Real-time PCR reveals a high incidence of Symbiodinium clade D at low levels in four scleractinian corals across the Great Barrier Reef: implications for symbiont shuffling. *Coral Reefs* 2007;26:449–57. 10.1007/s00338-007-0244-8

[47] Hoadley KD, Pettay DT, Grottoli AG, et al. High-temperature acclimation strategies within the thermally tolerant endosymbiont *Symbiodinium trenchii* and its coral host, *Turbinaria reniformis*, differ with changing pCO 2 and nutrients. *Mar Biol* 2016;163:134. 10.1007/s00227-016-2909-8

[48] Lee LK, Leaw CP, Lee LC, et al. Molecular diversity and assemblages of coral symbionts (Symbiodiniaceae) in diverse scleractinian coral species. *Mar Environ Res* 2022;179:105706. 10.1016/J.MARENVRES.2022.105706 35872442

[49] Grima AJ, Clases D, Gonzalez de Vega R, et al. Species-specific elementomes for scleractinian coral hosts and their associated Symbiodiniaceae. *Coral Reefs* 2022;41:1115–30. 10.1007/s00338-022-02259-2

[50] Ezzat L, Towle E, Irisson JO, et al. The relationship between heterotrophic feeding and inorganic nutrient availability in the scleractinian coral *T. reniformis* under a short-term temperature increase. *Limnol Oceanogr* 2016;61:89–102. 10.1002/lno.10200

[51] Hall ER, Muller EM, Goulet T, et al. Eutrophication may compromise the resilience of the Red Sea coral *Stylophora pistillata* to global change. *Mar Pollut Bull* 2018;131:701–11. 10.1016/j.marpolbul.2018.04.067 29886997

[52] Krueger T, Horwitz N, Bodin J, et al. Common reef-building coral in the Northern Red Sea resistant to elevated temperature and acidification. *R Soc Open Sci* 2017;4:170038. 10.1098/rsos.170038 PMC5451809 28573008

[53] Ezzat L, Maguer JF, Grover R, et al. Nutrient starvation impairs the trophic plasticity of reef-building corals under ocean warming. *Funct Ecol* 2019;33:643–53. 10.1111/1365-2435.13285

[54] R Core Team . R: A Language and Environment for Statistical Computing. R Foundation for Statistical Computing, Vienna, Austria. 2018. https://www.R-project.org/

[55] Dang KV, Pierangelini M, Roberty S, et al. Alternative photosynthetic electron transfers and bleaching phenotypes upon acute heat stress in *Symbiodinium* and *Breviolum* spp. (Symbiodiniaceae) in culture. *Front Mar Sci* 2019;6:479522. 10.3389/fmars.2019.00656

[56] Roberty S, Bailleul B, Berne N, et al. PSI Mehler reaction is the main alternative photosynthetic electron pathway in *Symbiodinium* sp., symbiotic dinoflagellates of cnidarians. *New Phytol* 2014;204:81–91. 10.1111/nph.12903 24975027

[57] Müller WEG, Seibert G, Beyer R, et al. Effect of cordycepin on nucleic acid metabolism in L5178Y cells and on nucleic acid-synthesizing enzyme systems. *Cancer Res* 1977;37:3824–33. 332340

[58] Sobell HM . Actinomycin and DNA transcription. *Proc Natl Acad Sci* 1985;82:5328–31. 10.1073/PNAS.82.16.5328 PMC390561 2410919

[59] Schneider-Poetsch T, Ju J, Eyler DE, et al. Inhibition of eukaryotic translation elongation by cycloheximide and lactimidomycin. *Nat Chem Biol* 2010;6:209–17. 10.1038/nchembio.304 PMC2831214 20118940

[60] Brodie JE, Kroon FJ, Schaffelke B, et al. Terrestrial pollutant runoff to the Great Barrier Reef: an update of issues, priorities and management responses. *Mar Pollut Bull* 2012;65:81–100. 10.1016/j.marpolbul.2011.12.012 22257553

[61] Radice VZ, Hoegh-Guldberg O, Fry B, et al. Upwelling as the major source of nitrogen for shallow and deep reef-building corals across an oceanic atoll system. *Funct Ecol* 2019;33:1120–34. 10.1111/1365-2435.13314

[62] Kopp C, Pernice M, Domart-Coulon I, et al. Highly dynamic cellular-level response of symbiotic coral to a sudden increase in environmental nitrogen. *MBio* 2013;4:e00052–13. 10.1128/mBio.00052-13 PMC3656441 23674611

[63] Miniview BJ . Algal nitrate reductases. *Eur J Phycol* 1997;32:3–8. 10.1080/09541449710001719315

[64] Taguchi S, Kinzie RA. Growth of zooxanthellae in culture with two nitrogen sources. *Mar Biol* 2001;138:149–55. 10.1007/s002270000435

[65] Maguer JF, L'Helguen S, Madec C, et al. Nitrogen uptake and assimilation kinetics in *Alexandrium minutum* (dynophyceae): effect of N-limited growth rate on nitrate and ammonium interactions. *J Phycol* 2007;43:295–303. 10.1111/j.1529-8817.2007.00334.x

[66] Hipkin CR, Al-Bassam BA, Syrett PJ. The roles of nitrate and ammonium in the regulation of the development of nitrate reductase in *Chlamydomonas reinhardii*. *Planta* 1980;150:13–8. 10.1007/BF00385608 24306519

[67] Lin S . Genomic understanding of dinoflagellates. *Res Microbiol* 2011;162:551–69. 10.1016/J.RESMIC.2011.04.006 21514379

[68] Roy S, Morse D. Transcription and maturation of mRNA in dinoflagellates. *Microorganisms* 2013;1:71–99. 10.3390/microorganisms1010071 PMC5029490 27694765

[69] Wisecaver JH, Hackett JD. Dinoflagellate genome evolution. *Ann Rev Microbiol* 2011;65:369–87. 10.1146/annurev-micro-090110-102841 21682644

[70] Zhang H, Hou Y, Miranda L, et al. Spliced leader RNA trans-splicing in dinoflagellates. *Proc Natl Acad Sci USA* 2007;104:4618–23. 10.1073/pnas.0700258104 PMC1838650 17360573

[71] Baumgarten S, Bayer T, Aranda M, et al. Integrating microRNA and mRNA expression profiling in *Symbiodinium microadriaticum*, a dinoflagellate symbiont of reef-building corals. *BMC Genomics* 2013;14:704. 10.1186/1471-2164-14-704 PMC3853145 24119094

[72] Zhu Y, Liao X, Han T, et al. Symbiodiniaceae microRNAs and their targeting sites in coral holobionts: a transcriptomics-based exploration. *Genomics* 2022;114:110404. 10.1016/J.YGENO.2022.110404 35714829

[73] Maor-Landaw K, van Oppen MJH, McFadden GI. Symbiotic lifestyle triggers drastic changes in the gene expression of the algal endosymbiont *Breviolum minutum* (Symbiodiniaceae). *Ecol Evol* 2020;10:451–66. 10.1002/ECE3.5910 PMC6972872 31993121

[74] Liew YJ, Li Y, Baumgarten S, et al. Condition-specific RNA editing in the coral symbiont *Symbiodinium microadriaticum*. *PLoS Genet* 2017;13:e1006619. 10.1371/JOURNAL.PGEN.1006619 PMC5357065 28245292

[75] Lillo C, Meyer C, Lea US, et al. Mechanism and importance of post-translational regulation of nitrate reductase. *J Exp Bot* 2004;55:1275–82. 10.1093/JXB/ERH132 15107452

[76] Kaiser WM, Weiner H, Kandlbinder A, et al. Modulation of nitrate reductase: some new insights, an unusual case and a potentially important side reaction. *J Exp Bot* 2002;53:875–82. 10.1093/JEXBOT/53.370.875 11912230

[77] Campbell WH . Nitrate reductase structure, function and regulation: bridging the gap between biochemistry and physiology. *Annu Rev Plant Biol* 1999;50:277–303. 10.1146/annurev.arplant.50.1.277 15012211

[78] Lillo C . Signalling cascades integrating light-enhanced nitrate metabolism. *Biochem J* 2008;415:11–9. 10.1042/BJ20081115 18778247

[79] Giordano M, Chen YB, Koblizek M, et al. Regulation of nitrate reductase in *Chlamydomonas reinhardtii* by the redox state of the plastoquinone pool. *Eur J Phycol* 2005;40:345–52. 10.1080/09670260500334263

[80] Chow F, Pedersén M, Oliveira MC. Modulation of nitrate reductase activity by photosynthetic electron transport chain and nitric oxide balance in the red macroalga *Gracilaria chilensis* (Gracilariales, Rhodophyta). *J Appl Phycol* 2013;25:1847–53. 10.1007/s10811-013-0005-8

[81] Nemie-Feyissa D, Królicka A, Førland N, et al. Post-translational control of nitrate reductase activity responding to light and photosynthesis evolved already in the early vascular plants. *J Plant Physiol* 2013;170:662–7. 10.1016/J.JPLPH.2012.12.010 23395536

[82] Provan F, Lillo C. Photosynthetic post-translational activation of nitrate reductase. *J Plant Physiol* 1999;154:605–9. 10.1016/S0176-1617(99)80233-8

[83] Hipkin CR, Thomas RJ, Syrett PJ. Effects of nitrogen deficiency on nitrate reductase, nitrate assimilation and photosynthesis in unicellular marine algae. *Mar Biol* 1983;77:101–5. 10.1007/BF00396306

[84] Basra AS, Dhawan AK, Goyal SS. DCMU inhibits in vivo nitrate reduction in illuminated barley (C3) leaves but not in maize (C4): a new mechanism for the role of light? *Planta* 2002;215:855–61. 10.1007/s00425-002-0802-9 12244452

[85] Sherameti I, Sopory SK, Trebicka A, et al. Photosynthetic electron transport determines nitrate reductase gene expression and activity in higher plants. *J Biol Chem* 2002;277:46594–600. 10.1074/jbc.M202924200 12244040

[86] Chow F, De Oliveira MC. Rapid and slow modulation of nitrate reductase activity in the red macroalga *Gracilaria chilensis* (Gracilariales, Rhodophyta): influence of different nitrogen sources. *J Appl Phycol* 2008;20:775–82. 10.1007/s10811-008-9310-z

[87] Zaheri B, Morse D. An overview of transcription in dinoflagellates. *Gene* 2022;829:146505. 10.1016/J.GENE.2022.146505 35447242

[88] Xiang T, Nelson W, Rodriguez J, et al. *Symbiodinium* transcriptome and global responses of cells to immediate changes in light intensity when grown under autotrophic or mixotrophic conditions. *Plant J* 2015;82:67–80. 10.1111/tpj.12789 25664570

[89] Krueger T, Horwitz N, Bodin J, et al. Intracellular competition for nitrogen controls dinoflagellate population density in corals. *Proc R Soc B Biol Sci* 2020;287:20200049. 10.1098/rspb.2020.0049 PMC7126079 32126963

[90] Baker DM, Andras JP, Jordán-Garza AG, et al. Nitrate competition in a coral symbiosis varies with temperature among *Symbiodinium* clades. *ISME J* 2013;7:1248–51. 10.1038/ismej.2013.12 PMC3660672 23407311

[91] Wong JCY, Enríquez S, Baker DM. Towards a trait-based understanding of Symbiodiniaceae nutrient acquisition strategies. *Coral Reefs* 2021;40:625–39. 10.1007/s00338-020-02034-1

[92] Martinez S, Grover R, Baker DM, et al. Symbiodiniaceae are the first site of heterotrophic nitrogen assimilation in reef-building corals. *MBio* 2022;13:e01601–22. 10.1128/MBIO.01601-22 PMC9600528 36125280

[93] Ezzat L, Fine M, Maguer JF, et al. Carbon and nitrogen acquisition in shallow and deep holobionts of the scleractinian coral *S. pistillata*. *Front Mar Sci* 2017;4:102. 10.3389/fmars.2017.00102

[94] Lyndby NH, Rädecker N, Bessette S, et al. Amoebocytes facilitate efficient carbon and nitrogen assimilation in the Cassiopea-Symbiodiniaceae symbiosis. *Proc R Soc B* 2020;287:20202393. 10.1098/RSPB.2020.2393 PMC7779505 33323078

[95] Röthig T, Puntin G, Wong JCY, et al. Holobiont nitrogen control and its potential for eutrophication resistance in an obligate photosymbiotic jellyfish. *Microbiome* 2021;9:127. 10.1186/s40168-021-01075-0 PMC8173792 34078452

[96] Krueger T, Bodin J, Horwitz N, et al. Temperature and feeding induce tissue level changes in autotrophic and heterotrophic nutrient allocation in the coral symbiosis – a NanoSIMS study. *Sci Rep* 2018;8:12710. 10.1038/s41598-018-31094-1 PMC6107511 30140050

[97] Rädecker N, Pogoreutz C, Gegner HM, et al. Heat stress destabilizes symbiotic nutrient cycling in corals. *Proc Natl Acad Sci USA* 2021;118:e2022653118. 10.1073/pnas.2022653118 PMC7865147 33500354

[98] Mayfield AB, Aguilar C, Kolodziej G, et al. Shotgun proteomic analysis of thermally challenged reef corals. *Front Mar Sci* 2021;8:660153. 10.3389/fmars.2021.660153

[99] Medrano E, Merselis DG, Bellantuono AJ, et al. Proteomic basis of symbiosis: a heterologous partner fails to duplicate homologous colonization in a novel cnidarian-symbiodiniaceae mutualism. *Front Microbiol* 2019;10:441065. 10.3389/fmicb.2019.01153 PMC6554683 31214134

[100] Camp EF, Kahlke T, Signal B, et al. Proteome metabolome and transcriptome data for three Symbiodiniaceae under ambient and heat stress conditions. *Sci Data* 2022;9:153. 10.1038/s41597-022-01258-w PMC8983644 35383179

[101] Oakley CA, Newson GI, Peng L, et al. The *Symbiodinium* proteome response to thermal and nutrient stresses. *Plant Cell Physiol* 2023;64:433–47. 10.1093/pcp/pcac175 PMC10109209 36565060

[102] Jiang J, Wang A, Deng X, et al. How Symbiodiniaceae meets the challenges of life during coral bleaching. *Coral Reefs* 2021;40:1339–53. 10.1007/s00338-021-02115-9

[103] Perez-Riverol Y, Bandla C, Kundu DJ, et al. The PRIDE database at 20 years: 2025 update. *Nucleic Acids Res* 2025;53:D543–53. 10.1093/nar/gkae1011 PMC11701690 39494541

