# Supplementary material for: Nitrogen-dependent regulation of nitrate reductase in coral symbioses
Source: ISME J. 2026 Oct 8;20(1):wrag260. doi: 10.1093/ismejo/wrag260 (PMC13685774; doi:10.1093/ismejo/wrag260)
Supplement: Supplementary file 1 [file supplementary_material_wrag260.zip › Supplementary_Material.docx]

**Supporting Information**

**S1. Supplementary material and methods.** Identification of Symbiodiniaceae strain identities through ITS2 rRNA gene sequencing.

**Table S2.** Composition of f/2 medium for the different N treatments.

**Table S3.** Composition of trace metal stock solution.

**Table S4.** Composition of vitamin solution.

**Figure S5.** Western blot analysis showing the failure to detect NR protein from N-replete SSB01 cultures.

**S6. Supplementary material and methods.** Analysis and quantification of immunoblot bands using the iBright analysis software (Thermo Scientific, USA).

**S7.** **Supplementary material, methods, and results.** Validation of the nitrate reductase immunoreactive band by LC-MS/MS.

**Table S7.** LC-MS/MS identification of the nitrate reductase candidate in two independent *B. minutum* protein databases.

**Figure S7.** Mapping of LC-MS/MS-identified peptides onto nitrate reductase candidate protein sequences from two independent *B. minutum* databases

**S8. Supplementary material, methods, and results.** Peptide competition assay.

**Figure S8.** Peptide competition assay assessing the specificity of nitrate reductase immunodetection in *B. minutum*

**S9.** **Supplementary material and methods.** Selection of primers for housekeeping genes.

**Table S9.** List of tested HKGs with references from the literature

**S10. Supplementary material and methods.** Quantification of *NR* gene expression from Ct values.

**S11. Supplementary material and methods.** *In hospite* experimental design and set up.

**Figure S12.** Western blot analysis showing failure to detect NR protein in FIS from N-replete corals.
