# Supplementary material for: Nitrogen-dependent regulation of nitrate reductase in coral symbioses
Source: ISME J. 2026 Oct 8;20(1):wrag260. doi: 10.1093/ismejo/wrag260 (PMC13685774; doi:10.1093/ismejo/wrag260)
Supplement: Supplementary file 1 [file supplementary_material_wrag260.zip › Stevenne_Supplementary_Material_wrag260.pdf]

Supporting Information

Nitrogen-Dependent Regulation of Nitrate Reductase in Coral Symbioses

Chloé Stévenne, Christine Ferrier-Pagès, Renaud Grover, Jean-Christophe Plumier, Stéphane Roberty

S1. Supplementary material and methods.

Identification of Symbiodiniaceae culture strain identities through ITS2 rRNA gene sequencing.

Symbiodiniaceae strain identities were confirmed by ITS2 rRNA gene sequencing. DNA was extracted from algae culture cell pellets using the DNeasy Plant Mini Kit (QIAGEN) following the manufacturer's instructions and by disrupting the cells in AP1 buffer using acid-washed glass beads (710-1180 µm, Sigma-Aldrich) and a TissueLyser II (QIAGEN) set to 30 Hz for 5 min. DNA concentration and purity were quantified by absorbance measurement on a Synergy Mx Biotek Plate Reader using a Take3 Microvolume Plate (Agilent, USA). The Symbiodiniaceae ITS2 rRNA gene was amplified using forward primer SYM_VAR_5.8S2 5'-GAATTGCAGAACTC-CGTGAACC-3' and reverse primer SYM_VAR_REV 5'-CGGGTTCWCTTGTYTGACTTCATGC-3' [1]. Between 5 and 10 ng of template DNA were used for PCR reaction using the Phusion High-Fidelity PCR Master Mix (Thermo Scientific) with initial denaturing at 98°C for 2 min followed by 35 cycles of 98°C for 10 sec, 56°C for 30 sec and 72°C for 30 sec, and finishing with a final extension at 72°C for 5 min. PCR amplification products were checked on a 2% agarose gel and concentrations were measured on the Synergy Mx before 15 µL of sample at 5 ng/µL were prepared for sequencing using the Mix2Seq kit and Mix2Seq overnight Sanger sequencing services (Eurofins, Germany). Resulting ITS2 sequences were locally blasted on the GeoSymbio database [2] using the NCBI blastn tool [3]. In this way, SSB01 cultures were confirmed as a B1 Symbiodiniaceae strain, corresponding to *Breviolum minutum* (blast score: 481, E-value: 4^{-138}), and the cultures isolated from *S. pistillata* were confirmed as A1 strain, corresponding to *Symbiodinium microadriaticum* (blast score: 431, E-value: 3^{-123}).

Identification of *in hospite* Symbiodiniaceae identities through ITS2 rRNA gene sequencing

DNA was extracted from FIS pellets, and the ITS2 rRNA gene amplified following the same protocol as for the Symbiodiniaceae cultures. ITS2 amplicons were sent to GIGA Genomics (ULiège, Belgium) for clean-up, library preparation, and sequencing on a MiSeq System using the Reagent Kit v3 for 2x250 bp paired-end reads (Illumina, USA). Demultiplexed forward and reverse reads were submitted to SymPortal [4] for remote analysis. The relative abundance of ITS2 type profiles was determined for each sample and assigned to the corresponding Symbiodiniaceae genera (*Symbiodinium*, *Cladocopium*, and *Durusdinium*).

Table S2. Composition of f/2 medium for the different N treatments.

Component	Stock Solution	Quantity in 1L ASW	Final Molar Concentration
Base for all f/2 media (corresponding to composition of f/2 NF medium)			
NaH_2PO_4 H_20	5 g/L diH_2O	1 mL	36.3 µM
Trace metal solution	[see S2 for composition]	1 mL	–
Vitamin solution	[see S3 for composition]	0.5 mL	–
Addition for f/2 NO_3^- 500 µM medium			
$NaNO_3$	75 g/L diH_2O	0.570 mL	500 µM
Addition for f/2 NH_4^+ 500 µM medium			
NH_4Cl	26.7 g/L	1 mL	500 µM

Table S3. Composition of trace metal stock solution.

Component	Stock Solution	Quantity in 1L ASW	Final Molar Concentration
$FeCl_3$ $6H_2O$	–	3.15 g	1.17×10^{-5} M
Na_2EDTA $2H_2O$	–	4.36 g	1.17×10^{-5} M
$CuSO_4$ $5H_2O$	9.8 g/L diH_2O	1 mL	3.93×10^{-8} M
Na_2MoO_4 $2H_2O$	6.3 g/L diH_2O	1 mL	2.60×10^{-8} M
$ZnSO_4$ $7H_2O$	22.0 g/L diH_2O	1 mL	7.65×10^{-8} M
$CoCl_2$ $6H_2O$	10.0 g/L diH_2O	1 mL	4.20×10^{-8} M
$MnCl_2$ $4H_2O$	180. 0 g/L diH_2O	1 mL	9.10×10^{-7} M

Table S4. Composition of vitamin solution.

Component	Stock Solution	Quantity in 1L ASW	Final Molar Concentration
Thiamine HCl (vit. B1)	–	200 mg	2.96×10^{-7} M
Biotin (vit. H)	0.1 g/L diH_2O	10 mL	2.05×10^{-9} M
Cyanocobalamin (vit. B_{12})	1.0 g/L diH_2O	1 mL	3.69×10^{-10} M

Composition of f/2 detailed in table S1, S2 and S3 are obtained from the literature [5, 6].

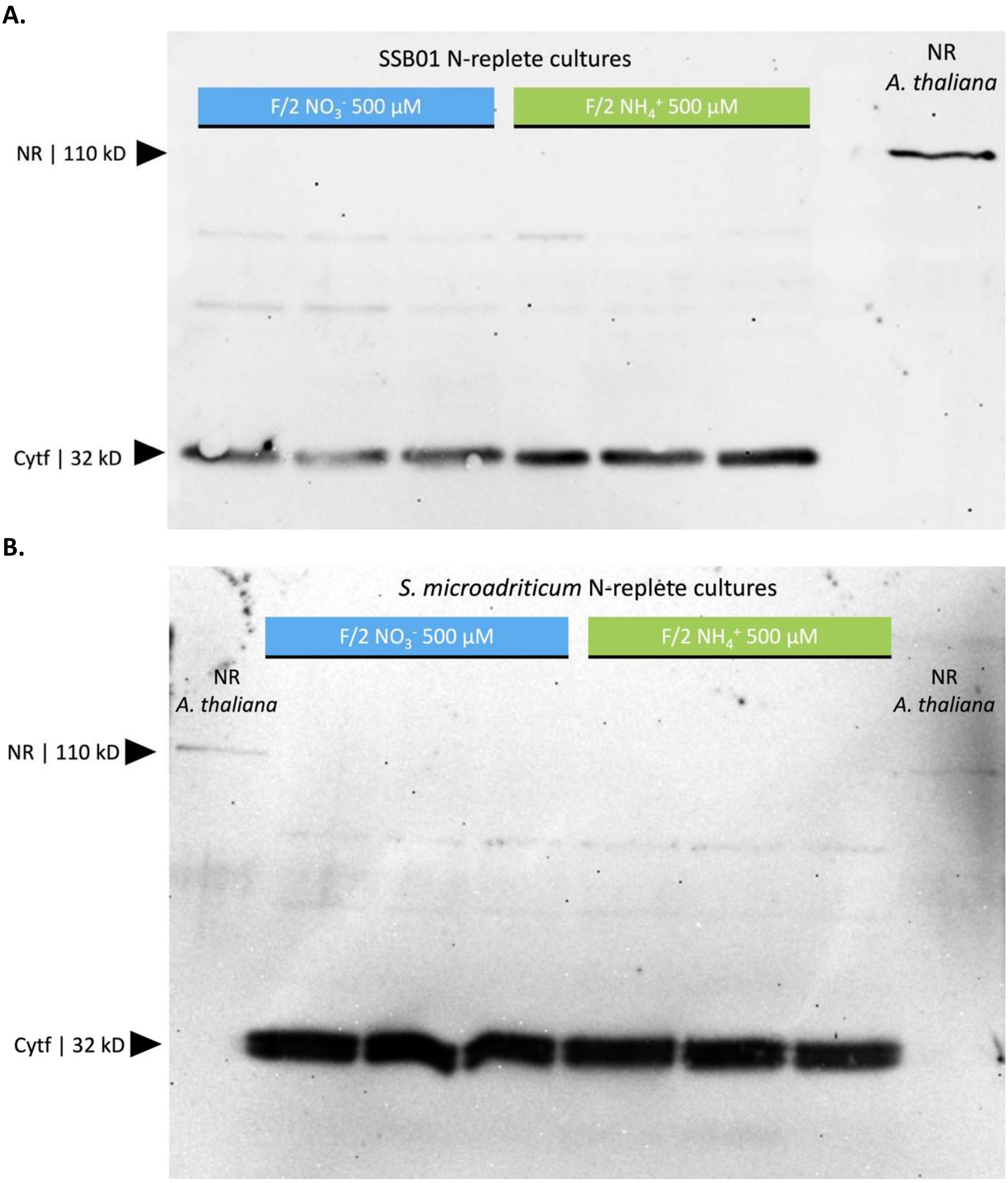

Figure S5. Western blot analysis showing the failure to detect NR protein from N-replete Symbiodiniaceae cultures. Proteins were extracted from N-replete *B. minutum* SSB01(A) and *S. microadriaticum* RSStyP-A (B) grown and maintained on the long-term in f/2 medium with 500 µM of either NO_3^- (blue) or NH_4^+ (green). Western blotting for the detection of the NR protein was conducted. The NR protein signal was below detection limits in all of the N-replete algae protein samples as illustrated on the western blot images with top row at size ~110 kDa corresponding to NR protein, and a bottom row at size ~32 kDa corresponding to the loading control (protein Cytf). A purified NR from *A. thaliana* served as a positive control.

S6. Supplementary material and methods. Analysis and quantification of immunoblot bands using the iBright analysis software (Thermo Scientific, USA).

Immunoblot band analysis was performed using the iBright analysis software (Thermo Scientific, USA). For nitrate reductase, an immunoreactive band was consistently detected at approximately 110 kDa, migrating slightly faster than the purified *A. thaliana* nitrate reductase, but within the expected apparent molecular weight range of eukaryotic nitrate reducases [7, 8]. When additional migrating bands were observed, only the consistently detected band in the ~110-120 kDa range was selected for analysis. In rare cases where two closely migrating bands were present within this molecular weight range (< 5 kDa difference), both bands were included within the same selected area for quantification.

The area of the bands corresponding to nitrate reductase (110 kDa) and cytochrome f (32 kDa) were manually selected, and the volume of each band, corresponding to the sum of each pixel grayscale intensity in the region of the band, was measured and corrected for local background noise (local bg. corr. vol.) by the software. Nitrate reductase protein expression was calculated as the ratio between NR band and Cytf band signal expressed as local background corrected volume.

S7. Supplementary material, methods and results. Validation of the NR immunoreactive band by LC/MS-MS**.**

SDS-PAGE, gel excision and in-gel digestion

To assess the identity of the immunoreactive band detected with the anti-NR antibody, 50 µg of protein extracts from four independent *B. minutum* cultures were separated by SDS-PAGE in parallel with samples analysed by western blotting. For each independent gel, the 100-120 kDa region corresponding to the principal immunoreactive band was excised for mass spectrometry analysis. Protein bands were visualized by colloidal Coomassie Blue staining, reduced with DTT, alkylated with chloroacetamide, and subjected to in-gel digestion with trypsin. Peptides were extracted with 0.1% trifluoroacetic acid (TFA) in 65% acetonitrile (ACN) and dried in a SpeedVac. The excised gel region from each of the four cultures was analysed independently by LC-MS/MS.

LC-MS/MS analysis

Peptides were dissolved in solvent A (0.1% TFA in 2% ACN), directly loaded onto a reversed-phase pre-column (Acclaim PepMap 100, Thermo Scientific), and eluted in backflush mode. Peptide separation was performed using a reversed-phase analytical column (Acclaim PepMap RSLC, 0.075 × 250 mm, Thermo Scientific) on a Vanquish Neo system. Peptides were separated at a constant flow rate of 300 nL min^{-1} using a linear gradient of 4-27.5% solvent B (0.1% formic acid in 98% ACN) for 40 min, followed by 27.5-50% solvent B for 20 min and 50-95% solvent B for 10 min, with the column maintained at 95% solvent B for a final 10 min. Peptides were analysed using an Orbitrap Fusion Lumos Tribrid mass spectrometer (Thermo Fisher Scientific). The peptides were subjected to NSI source followed by tandem mass spectrometry (MS/MS) in Fusion Lumos coupled online to the nano-LC. Intact peptides were detected in the Orbitrap at a resolution of 120,000. Peptides were selected for MS/MS using HCD setting at 30, ion fragments were detected in the linear ion trap (LUMOS). A data-dependent procedure that alternated between one MS scan followed by MS/MS scans was applied for 3 seconds for ions above a threshold ion count of 2.0E4 in the MS survey scan with 40.0s dynamic exclusion. The electrospray voltage applied was 2.1 kV. MS1 spectrum was obtained with an AGC target of 4E5 ions and a maximum injection time of 50 ms, and MS2 spectra were acquired with an AGC target of 5E4 ions and a maximum injection time set to dynamic. For MS scans, the m/z scan range was 375 to 1800.

Protein database searching and identification

Raw MS/MS data were processed using the Sequest HT search engine implemented in Proteome Discoverer 3.1 SP1 (Thermo Fisher Scientific). Because curated reference proteomes do not exist for non-model Symbiodiniaceae and sequence-database

completeness can be limiting , the MS/MS data were searched independently against two *B. minutum*-related protein sequence databases: (i) a genome-derived predicted protein set from the draft nuclear genome of *B. minutum* strain Mf1.05b [9]and (ii) a custom protein database constructed by Mashini et al. [10, 11] from a *B. minutum* transcriptome [12]. Candidate NR sequences in these databases were identified by BLASTX of NR-predicted nucleotide sequences from transcriptomic studies [13, 14] and whole genome shotgun sequencing [15] of several Symbiodiniaceae species. Additionally, BLASTP was conducted using reference NR protein sequences from several algae and plant species to confirm the identity of the candidate NR proteins. All sequence data and BLAST results are available in the Zenodo repository: https://doi.org/10.5281/zenodo.18352345.

Trypsin was specified as the cleavage enzyme, allowing up to two missed cleavages, a maximum of four modifications per peptide, and precursor charge states up to +5. Precursor and fragment mass tolerances were set to 10 ppm and 0.1 Da, respectively. Oxidation of methionine (+15.995 Da), conversion of N-terminal glutamine to pyroglutamate (−17.027 Da), and conversion of N-terminal glutamate to pyroglutamate (−18.011 Da) were specified as variable modifications. Carbamidomethylation of cysteine (+57.021 Da) was specified as a fixed modification. False discovery rates (FDRs) were estimated using Percolator, with thresholds of 1% applied at the protein, peptide, and modification-site levels. Protein identification was evaluated based on protein-level confidence, the number of distinct and unique peptides, peptide-spectrum matches (PSMs), and protein sequence coverage. To assess the robustness of the protein assignment with respect to database choice, NR protein sequences and the corresponding identified peptides from the two independent database searches were compared directly.

Using the genome-based database [9], analysis of the 100-120 kDa gel band identified the nitrate reductase candidate protein (symbB.v1.2.036677.t1) with high confidence, based on 42 distinct peptides, including 33 unique peptides, corresponding to 43% sequence coverage and 231 peptide-spectrum matches (PSMs). Using the transcriptome-based database [13, 14], the NR candidate protein (m.62664) was identified with 35 peptides, including 32 unique peptides, corresponding to 42% sequence coverage and 203 peptide-spectrum matches (***Table S7, Figure S7***). Thirty-four distinct peptide sequences were shared between the two searches. These results confirm the presence of nitrate reductase in the gel region corresponding to the main western blot immunoreactive band.

Table S7. LC-MS/MS identification of the nitrate reductase candidate in two independent *B. minutum* protein databases. LC-MS/MS data from the 100-120 kDa immunoreactive gel region were searched independently against genome- and transcriptome-derived *B. minutum* databases. The NR candidate protein was identified with high confidence in both databases, based on the number of distinct and unique peptides, peptide-spectrum matches (PSMs), and sequence coverage.

Parameter	Genome-based DB (Shoguchi et al. 2013)	Transcriptome-based DB (Mashini et al. 2023, 2024)
**Protein accession**	symbB.v1.2.036677.t1	comp44060_c0::comp44060_c0_seq4::g.62664::m.62664
**Identification confidence**	High	High
**Protein length**	1197 aa	934 aa
**Predicted molecular weight**	131.9 kDa	103.4 kDa
**Distinct peptides**	42	35
**Unique peptides**	33	32
**Sequence coverage**	43%	42%
**Peptide-spectrum matches (PSMs)**	231	203

A **Genome-derived NR candidate**

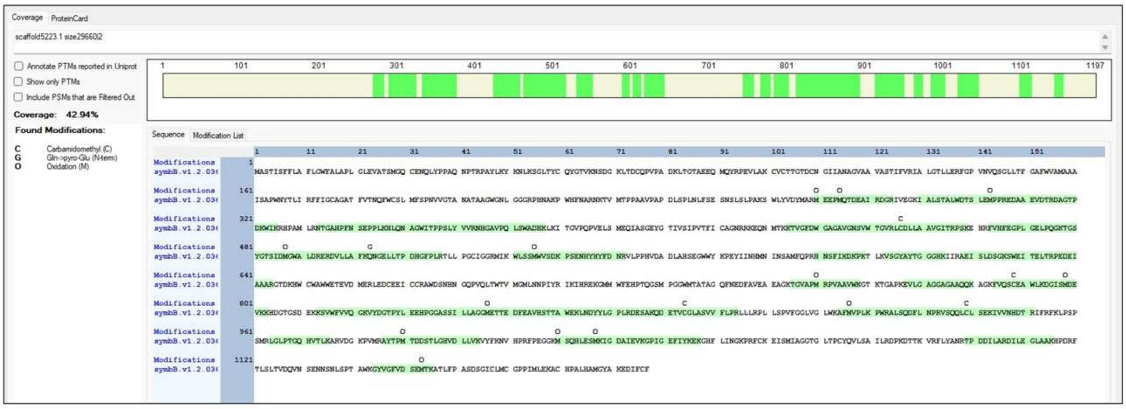

B **Transcriptome-derived NR candidate**

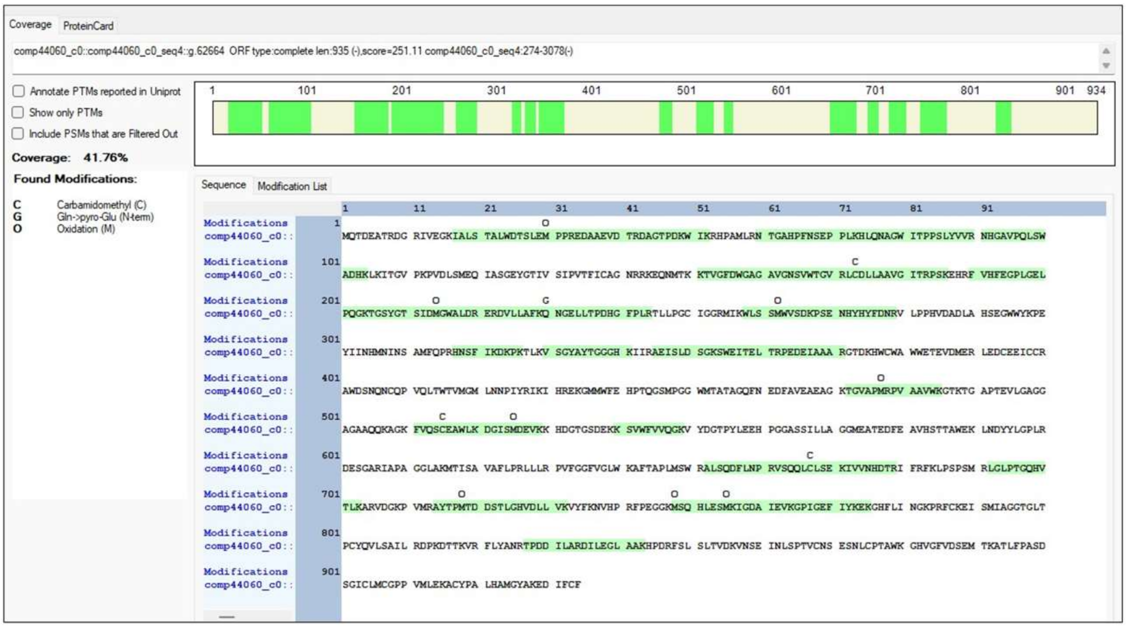

Figure S7. Mapping of LC-MS/MS-identified peptides onto nitrate reductase candidate protein sequences from two independent *B. minutum* databases. LC-MS/MS data obtained from the 100-120 kDA immunoreactive gel region were searched independently against (A) a genome-derived and (B) a transcriptome-derived *B. minutum* protein database. Identified peptides are highlighted in green on the predicted nitrate reductase sequences, accounting for 43% and 42% sequence coverage respectively. Despite the differences in predicted protein length and sequence between the two database-derived candidates, the identified peptides mapped to corresponding regions of both sequences.

S8. Supplementary material, methods and results. Peptide competition assay

Antibody recognition was evaluated by peptide competition using an aliquot of the authentic immunizing peptide supplied directly by Agrisera for antibody AS08 310. Protein extracts from two independent cultures of *B. minutum* were loaded at 25 µg total protein per lane. Recombinant *A. thaliana* nitrate reductase expressed in *Pichia pastoris* (N0163; Sigma-Aldrich) was included as a positive control and loaded at 0.1 and 1 µg per lane. Proteins were separated by SDS-PAGE and transferred onto PVDF membranes under the conditions described in the main text. Membranes were blocked for 1 h at room temperature in blocking solution (5% non-fat dry milk in TBS-T). Two otherwise identical primary-antibody solutions were prepared in parallel. For the competition condition, the anti-NR antibody was diluted 1:1000 in TBS-T 2% milk and pre-incubated with the manufacturer-supplied immunizing peptide at a final concentration of 2.67 µM for 1 h at room temperature, with gentle agitation (peptide:antibody molar ratio of 100:1). For the control condition, the antibody was diluted identically and incubated for the same duration and at the same temperature in the absence of peptide. Duplicate membranes were incubated with either the untreated antibody or the peptide-pre-incubated antibody overnight at 4°C. Membranes were subsequently washed 6 times for 5 min in TBS-T and incubated with HRP-conjugated goat anti-rabbit secondary antibodies (Agrisera AS09 602), diluted 1:25000 in 2% non-fat dry milk in TBS-T, for 1 h at room temperature. After 6 additional washes, immunoreactive signals were detected using Amersham ECL Prime detection system (Cytiva, USA) and acquired with an iBright FL1000 imaging system (Thermo Scientific, USA). Images obtained under the two conditions were acquired simultaneously and processed using identical acquisition and display settings. Signal inhibition following antibody pre-incubation with the authentic immunizing peptide was assessed qualitatively by comparing recognition of recombinant *A. thaliana* NR and the corresponding immunoreactive species in *B. minutum* extracts between the control and peptide-competition conditions.

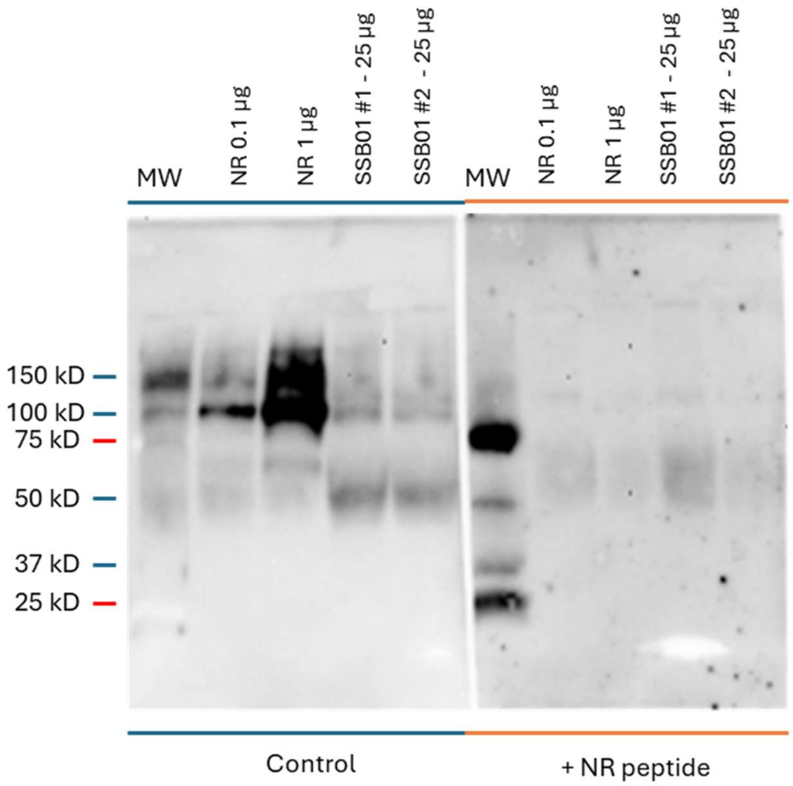

Figure S8. Peptide competition assay assessing the specificity of nitrate reductase immunodetection in *B. minutum*. Representative western blot showing recognition of recombinant *A. thaliana* NR and protein extracts from two independent *B. minutum* SSB01 cultures by the Agrisera AS08 310 antibody under control conditions or following pre-incubation with the authentic manufacturer-supplied immunizing peptide. Lane labels indicate the amounts of recombinant NR or total *B. minutum* protein loaded. Signal inhibition following antibody pre-incubation with the authentic immunizing peptide was assessed qualitatively by comparing recognition of recombinant *A. thaliana* NR and the corresponding immunoreactive species in *B. minutum* extracts between the control and peptide-competition conditions. Peptide competition markedly reduced recognition of recombinant NR and the principal immunoreactive species in the *B. minutum* extracts.

S9. Supplementary material and methods. Design of qRT-PCR primers

Selection of primers for housekeeping genes

Several primer pairs for housekeeping genes (HKG) were selected from the literature (table S9) [16–19] and tested using a PCR profile consisting of an initial denaturation of 2 min at 94°C, followed by 35 cycles of 30 s at 94°C, 30 s at 55°C and 30 s at 72°C, with a final elongation of 7 min at 72°C, using GoTaq Green Master Mix (Promega, USA). PCR amplification products were visualized on 2% agarose gels using GeneRuler 1kb Plus DNA ladder (Thermo Scientific, USA) for amplicon size estimation. Primers yielding successful amplifications on SSB01 cultures were selected and tested for stability using the geNorm analysis tool in Qbase+ [20, 21]. Based on this, primer pairs Cyc_Maru [19] and Act1_May [18] for genes *cyclophilin* and *actin1*, respectively, were selected as HKG for all experiments investigating *NR* gene expression levels.

Table S9. List of tested HKGs with references from the literature

Gene	Primer pair name	Target type	Forward primer (5'-3')	Reverse primer (5'-3')	Amplicon size	Amplification test	qPCR & geNorm test	Reference
Calmodulin	Cal_Kru	B1	TGATGGCGCGCAAGATGAAGG	TGCCATCGCGATCGAAAACCTTG	78 bp	Passed	Failed melt curve	(Krueger et al. 2015)
Cytochrome Oxidase Subunit 1	Cox_Kru	B1	TCTGTCTTCCTCTCACATCTCT	CCACTGCACCATTTCCAAGA	82 bp	Passed	Rejected by geNorm test	(Krueger et al. 2015)
S4 Ribosomal Protein	RpS4_Ros	C3	CCGCACAAACTGCGTGAGT	CGCTGCATGACGATCATCTT	101 bp	Passed	Failed melt curve	(Rosic et al. 2011)
Actin	Act1_May	B1	CACCACCATGTTCACAGGAA	AGCCACCACCTTGATCTTCA	87 bp	Passed	Passed	(Mayfield et al. 2014)
Cyclophilin	Cyc_Ros	C3	ATGTGCCAGGGTGGAGACTT	CCTGTGTGCTTCAGGGTGAA	101 bp	Passed	Failed melt curve	(Rosic et al. 2011)
Cyclophilin	Cyc_Maru	B1	TGCTTGAGGGTAAAGTTCTC	CAACCCCTTCATTTCAAAGG	N.A.	Passed	Passed	(Maruyama et al. 2022)

Investigation into NR primer pair specificity to putative *NR* coding sequencing

The primer pair NR_s6_34, originally designed by Xiang et al. (2020) and used by Maruyama et al. (2022), was carefully evaluated before its use in our study [14, 19]. PrimerBLAST confirmed perfect matching to the s6_34 transcript of SSB01. BLASTx analysis of s6_34 against the nr database (all non-redundant GenBank CDS) showed high-scoring hits to nitrate reductase [NADH] / NITA protein predictions from Symbiodiniaceae [22]. BLASTx against the curated SwissProt database further confirmed the identity of s6_34 as a canonical eukaryotic assimilatory nitrate reductase, with top hits to experimentally validated NR proteins from plants, green algae, fungi, and oomycetes [22]. Although the sequence identity was lower (~40%), as expected across deep evolutionary distances, protein length (~900) and domain architecture were conserved. Importantly, the qRT-PCR amplicon (nucleotides 1154-1271 of s6_34; amino acids ~385-424) corresponds to a conserved structural region immediately preceding the molybdopterin-binding catalytic domain.

S10. Supplementary material and methods. Quantification of *NR* gene expression from Ct values.

Ct values (threshold cycle with fluorescence threshold set to 0.2) of technical triplicates were averaged for each sample, and the Ct values of the two HKGs Cyc_Maru and Act1_May were averaged and subtracted from the mean Ct value of the sample to obtain ΔCt ($\Delta Ct = Ct_{sample} - (Ct_{Cyc\_Maru} + Ct_{Act1\_May})/2$). The ΔCt value was then subtracted from the mean ΔCt value of the samples at time 0 h to express relative expression ΔΔCt. Fold gene expression was calculated as $2^{-\Delta\Delta Ct}$ for each sample (biological replicate) in each experimental condition.

S11. Supplementary material and methods. *In hospite* experimental design and set up.

All coral colonies were maintained in flow-through seawater aquaria at the Centre Scientifique de Monaco (Monaco). One mother colony of each species was used to produce 36 nubbins which were evenly distributed and allowed to recover in two independent aquaria (28 L) supplied continuously with natural oligotrophic seawater (~0.5-0.7 µM DIN and ~0.2 µM DIP, see report below) at a flow rate of 20 L h^{-1}, pumped at a depth of 50 m. During the four weeks of recovery, nubbins were fed twice a week with *Artemia* nauplii, maintained at 25°C ± 0.5°C, with constant agitation provided by water pumps, and under 150 µmol photons m^{-2} s^{-1} in a 12h:12h day:night cycle. Feeding of the coral nubbins was stopped two weeks prior to the experiments to avoid interaction with the nitrogen enrichments.

All treatments were carried out in duplicate 28 L tanks under similar temperature (25°C ± 0.5°C), flow, and light (150 µmol photon m^{-2} s^{-1}) conditions as described above. For both experiments, coral nubbins were first incubated in nitrogen-depleted water for 24 h. Nutrient-depleted water was achieved using a bio-chemical filter (Fluidized bed filter BF-700, Otto, Australia) following a previously described and validated methodology, which reduced total inorganic nitrogen to ≤0.08 µM in the original validation study [23]. All experimental tanks were kept as a closed system and nitrate enrichment was achieved by mixing a $NaNO_3$ concentrated stock solution to the tank seawater just before coral nubbins were transferred to the experimental tanks. Treated nubbins were incubated in 50 µM nitrate-enriched FSW. This concentration, lower than that used in cultured Symbiodiniaceae experiments, was selected to induce a detectable NR response *in hospite* while minimizing potential stress to the coral holobiont under laboratory conditions.

For experiment B, symbionts were freshly isolated (FIS) from the coral host tissues using waterpick blasting with 0.2 µm filtered seawater (FSW). Coral tissue slurry was then homogenized using a syringe equipped with a 24G needle to dissociate symbiont cells from the coral tissue, followed by three centrifugation and wash steps to pellet symbiont cells and remove the supernatant. FIS were incubated in 200 mL culture flasks with ventilated cap, at a temperature of 25°C ± 0.5°C with gentle agitation using a magnetic stirrer and under 150 µmol photons m^{-2} s^{-1} constant light.

Experimental A2 Aquaria Nutrient Monitoring (April & May 2022)

Individual tank nutrient monitoring

	Tank	NO3 (µmol/L)	NH4 (µmol/L)	DIN (µmol/L)	PO4 (µmol/L)
Apr-22	A2-1	0.72	0.10	0.82	0.20
	A2-1	0.69	0.15	0.84	0.18
	A2-3	0.47	0.12	0.59	0.16
	A2-3	0.76	0.10	0.86	0.19
	A2-4	0.32	0.17	0.49	0.17
	A2-4	0.34	0.23	0.57	0.19
May-22	A2-1	0.33	0.37	0.70	0.27
	A2-1	0.27	0.19	0.46	0.29
	A2-3	0.36	0.45	0.81	0.14
	A2-3	0.23	0.1	0.33	0.26
	A2-4	0.12	0.24	0.36	0.11
	A2-4	NA	NA	NA	NA

Mean values nutrient monitoring

		NO3 (µmol/L)	NH4 (µmol/L)	DIN (µmol/L)	PO4 (µmol/L)
Apr-22	AVG	**0.55**	**0.15**	**0.70**	**0.18**
	SE	0.08	0.02	0.07	0.01
May-22	AVG	**0.26**	**0.27**	**0.53**	**0.21**
	SE	0.04	0.06	0.09	0.03
ALL	AVG	**0.42**	**0.20**	**0.62**	**0.20**
	SE	0.06	0.03	0.06	0.02

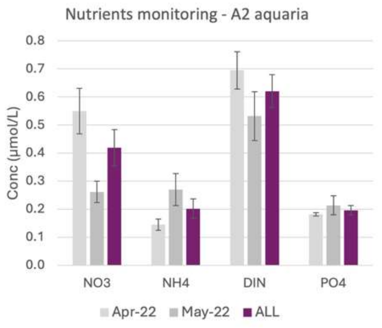

The aquaria system at the Centre Scientifique de Monaco is continuously supplied with natural oligotrophic seawater in an open-flow configuration. Long-term routine monitoring conducted at the facility indicates limited temporal fluctuations in nutrient concentrations, with typical values around ~0.5 µM DIN and ~0.2 µM DIP under non-enriched conditions [24–26]. Nutrient concentrations were not measured during the experimental period in March 2022 due to temporary unavailability of the nutrient autoanalyzer. Alternatively, the best available quantitative context is provided by monitoring data obtained in April and May 2022 from the same aquarium system and water supply configuration, which showed that nutrient levels remained within the typical recorded range.

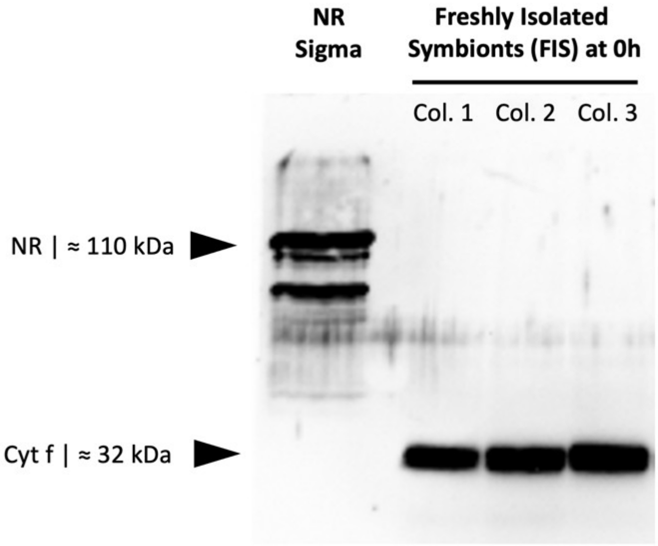

Figure S12. Western blot analysis showing failure to detect NR protein in FIS from N-replete corals. After incubation of N-replete *S. pistillata* corals in seawater enriched with NO_3^-, symbionts were isolated (FIS) and their proteins extracted for western blotting for the detection of the NR protein. The NR protein could not be detected in any of the FIS samples as illustrated on the image of one western blot membrane with top row at size ~110 kDa corresponding to NR protein, and a bottom row at size ~32 kDa corresponding to the loading control (protein Cytf). NR Sigma is a purified NR from *A. thaliana* serving as a positive control.

References

1. Hume BCC et al. An improved primer set and amplification protocol with increased specificity and sensitivity targeting the *Symbiodinium* ITS2 region. *PeerJ* 2018;**6**:e4816. https://doi.org/10.7717/peerj.4816
2. Franklin EC et al. GeoSymbio: A hybrid, cloud-based web application of global geospatial bioinformatics and ecoinformatics for *Symbiodinium*-host symbioses. *Mol Ecol Resour* 2012;**12**:369–373. https://doi.org/10.1111/j.1755-0998.2011.03081.x
3. NCBI. BLAST Command Line Applications User Manual. Bethesda (MD), 2008.
4. Hume BCC et al. SymPortal: A novel analytical framework and platform for coral algal symbiont next-generation sequencing ITS2 profiling. *Mol Ecol Resour* 2019;**19**:1063–1080. https://doi.org/10.1111/1755-0998.13004
5. Guillard RRL, Ryther JH. Studies of marine planktonic diatoms I. Cyclotella nana Hustedt and Detonula confervacea (Cleve) Gran. *Can J Microbiol* 1962;**8**:229–239. https://doi.org/10.1139/m62-029
6. Guillard RRL. Culture of phytoplankton for feeding marine invertebrates. In: Smith WL, Chanley MH (eds.), Culture of Marine Invertebrate Animals. Boston: Springer, 1975, 26–60.
7. Berges J. Miniview: Algal nitrate reductases. *Eur J Phycol* 1997;**32**:3–8. https://doi.org/10.1080/09541449710001719315
8. Sanz-Luque E et al. Understanding nitrate assimilation and its regulation in microalgae. *Front Plant Sci* 2015;**6**:899. https://doi.org/10.3389/fpls.2015.00899
9. Shoguchi E et al. Draft assembly of the *Symbiodinium minutum* nuclear genome reveals dinoflagellate gene structure. *Curr Biol* 2013;**23**:1399–1408. https://doi.org/10.1016/j.cub.2013.05.062
10. Mashini A et al. Proteomes of native and non-native symbionts reveal responses underpinning host-symbiont specificity in the cnidarian-dinoflagellate symbiosis. *ISME J* 2024;**18**:122. https://doi.org/10.1093/ismejo/wrae122
11. Mashini AG et al. The Influence of Symbiosis on the Proteome of the Exaiptasia Endosymbiont Breviolum minutum. *Microorganisms* 2023;**11**:292. https://doi.org/10.3390/microorganisms11020292
12. Parkinson JE et al. Gene expression variation resolves species and individual strains among coral-associated dinoflagellates within the genus symbiodinium. *Genome Biol Evol* 2016;**8**:665–680. https://doi.org/10.1093/gbe/evw019
13. Yuyama I et al. Transcriptomic changes with increasing algal symbiont reveal the detailed process underlying establishment of coral-algal symbiosis. *Sci Rep* 2018;**8**:16802. https://doi.org/10.1038/s41598-018-34575-5
14. Xiang T et al. Symbiont population control by host-symbiont metabolic interaction in Symbiodiniaceae-cnidarian associations. *Nat Commun* 2020;**11**:108. https://doi.org/10.1038/s41467-019-13963-z
15. Aranda M et al. Genomes of coral dinoflagellate symbionts highlight evolutionary adaptations conducive to a symbiotic lifestyle. *Sci Rep* 2016;**6**:39734. https://doi.org/10.1038/srep39734
16. Hawkins TD et al. Antioxidant responses to heat and light stress differ with habitat in a common reef coral. *Coral Reefs* 2015;**34**:1229–1241. https://doi.org/10.1007/s00338-015-1345-4

17. Rosic NN et al. Validation of housekeeping genes for gene expression studies in *Symbiodinium* exposed to thermal and light stress. *Mar Biotechnol* 2011;**13**:355–365. https://doi.org/10.1007/s10126-010-9308-9
18. Mayfield AB et al. Rubisco expression in the dinoflagellate *Symbiodinium* sp. is influenced by both photoperiod and endosymbiotic lifestyle. *Mar Biotechnol* 2014;**16**:371–384. https://doi.org/10.1007/s10126-014-9558-z
19. Maruyama S et al. Algae from *Aiptasia* egesta are robust representations of Symbiodiniaceae in the free-living state. *PeerJ* 2022;**10**:e13796. https://doi.org/10.7717/PEERJ.13796
20. Vandesompele J et al. Accurate normalization of real-time quantitative RT-PCR data by geometric averaging of multiple internal control genes. *Genome Biol* 2002;**3**:research0034. https://doi.org/10.1186/gb-2002-3-7-research0034
21. Hellemans J et al. qBase relative quantification framework and software for management and automated analysis of real-time quantitative PCR data. *Genome Biol* 2007;**8**:R19. https://doi.org/10.1186/gb-2007-8-2-r19
22. Altschul SF et al. Gapped BLAST and PSI-BLAST: a new generation of protein database search programs. *Nucleic Acids Res* 1997;**25**:3389–3402. https://doi.org/10.1093/nar/25.17.3389
23. Ezzat L et al. Nutrient starvation impairs the trophic plasticity of reef-building corals under ocean warming. *Funct Ecol* 2019;**33**:643–653. https://doi.org/10.1111/1365-2435.13285
24. Blanckaert ACA et al. Nutrient starvation and nitrate pollution impairs the assimilation of dissolved organic phosphorus in coral-Symbiodiniaceae symbiosis. *Sci Total Environ* 2023;**858**:159944. https://doi.org/10.1016/j.scitotenv.2022.159944
25. Blanckaert ACA et al. Low levels of ultra-violet radiation mitigate the deleterious effects of nitrate and thermal stress on coral photosynthesis. *Mar Pollut Bull* 2021;**167**:112257. https://doi.org/10.1016/j.marpolbul.2021.112257
26. Ezzat L et al. The relationship between heterotrophic feeding and inorganic nutrient availability in the scleractinian coral *T. reniformis* under a short-term temperature increase. *Limnol Oceanogr* 2016;**61**:89–102. https://doi.org/10.1002/lno.10200
